# Postnatal Azithromycin Is Neuroprotective and Anti-Inflammatory in a Piglet Model of Hypoxic-Ischemic Encephalopathy

**DOI:** 10.1161/STROKEAHA.125.054318

**Published:** 2026-02-13

**Authors:** Raymand Pang, Christopher Meehan, George Maple, Yujin Kim, Ilenia D’Angelo, Alison Mintoft, Francisco Torrealdea, Mariya Hristova, Manjunath P. Pai, John D.E. Barks, Nicola J. Robertson

**Affiliations:** Department of Neonatology, Institute for Women’s Health, University College London, United Kingdom (R.P., C.M., G.M., Y.K., I.D., A.M., M.H., N.J.R.).; Medical Physics and Biomedical Engineering, University College London Hospitals, United Kingdom (F.T.).; Department of Clinical Pharmacy, College of Pharmacy (M.P.P.), The University of Michigan, Ann Arbor.; Department of Pediatrics (J.D.E.B.), The University of Michigan, Ann Arbor.; National Institute for Health and Care Research (NIHR) University College London Hospitals Biomedical Research Centre, United Kingdom (N.J.R.).

**Keywords:** azithromycin, Escherichia coli, inflammation, ischemia, neuroprotection

## Abstract

**BACKGROUND:**

There is an urgent need for cytoprotective therapies for hypoxic-ischemic encephalopathy in low- and middle-income countries, where therapeutic hypothermia is not beneficial. Infection and inflammation are important risk factors for hypoxic-ischemic encephalopathy in sub-Saharan Africa, and immunomodulatory therapies might improve outcomes. Azithromycin is neuroprotective in rodent hypoxic-ischemic encephalopathy models but validation in large animal models is needed. Our aim was to assess safety and efficacy of intravenous azithromycin after inflammation-amplified hypoxia-ischemia in newborn piglets.

**METHODS:**

Twenty-five piglets underwent inflammation-amplified hypoxia-ischemia by bilateral carotid artery occlusion and reduction in FiO_2_ to 6% at 4 hours after the start of *Escherichia coli* liposaccharide infusion (2 mcg/kg bolus+1 mcg/kg per hour over 12 hours). At 1 hour after inflammation-amplified hypoxia-ischemia, piglets were randomized to receive vehicle (n=12) or azithromycin 20 mg/kg over 1 hour (n=13), repeated at 24 and 48 hours. Piglets underwent neurocritical care for 65 hours, including continuous amplitude-integrated encephalography, magnetic resonance imaging/magnetic resonance spectroscopy at 60 hours, and brain immunohistochemistry. Group differences were evaluated by Bayesian analysis with noninformative priors; the a priori threshold probability of superiority (Pr_[__sup]_) was set at 95%.

**RESULTS:**

Insult severity did not differ between groups. Plasma azithromycin peak concentration (C_max_) of ≈2 mg/mL and brain tissue concentrations of ≈1.5 mg/kg were achieved. Azithromycin was associated with improved amplitude-integrated encephalography background (Pr_[__sup]_=98.6%), an overall increase in neuronal nuclear antigen (NeuN+) cells (Pr_[__sup]_=97.8%), increase in Iba1 (ionized calcium-binding adapter molecule 1)+cells (Pr_[__sup]_=99.6%) and increase in Iba1 ramification index (resting microglial morphology; Pr_[__sup__]_=99.6%). The treatment benefit on magnetic resonance spectroscopy lactate to N-acetyl aspartate peak area ratio was modest (Pr_[__sup]_ of 82.7% and 68.5% in the thalamic and white matter voxels, respectively).

**CONCLUSIONS:**

Azithromycin, administered after inflammation-amplified hypoxia-ischemia was safe and associated with increased neuronal survival, microglial immunomodulation, enhanced amplitude-integrated encephalography recovery, and a modest benefit on lactate to N-acetyl aspartate peak area ratio. These safety and efficacy data of azithromycin as a monotherapy hold promise to improve outcomes for hypoxic-ischemic encephalopathy in low- and middle-income countries.

Hypoxic-ischemic encephalopathy (HIE) is a leading cause of global newborn mortality and morbidity, disproportionately affecting babies in low- and middle-income countries (LMICs) where >85% of all global cases of HIE occur.^1^

Although the benefit of therapeutic hypothermia (HT) for HIE in high-resource settings is well established, negative findings from a trial of HT for moderate to severe HIE in South Asia highlight the need for alternative therapies to improve outcomes in some LMICs.^2^ Several factors may contribute to the lack of benefit of HT in LMICs, including higher rates of perinatal infection and inflammation,^3^ lower rates of sentinel events, poor nutrition, and chronic in utero hypoxia-ischemia (HI). Repurposing azithromycin, a macrolide antibiotic, is an attractive option given its established safety in mass drug administration campaigns to reduce childhood mortality.^4^ Several clinical trials are underway in sub-Saharan Africa to reduce maternal and neonatal mortality (URL: https://www.clinicaltrials.gov; Unique identifiers: NCT03871491 and NCT03909737). In high-resource settings, intravenous azithromycin has demonstrated safety in clinical trials for preterm infants to reduce the risk of bronchopulmonary dysplasia; however, data on the effect of azithromycin on corrected QT interval (QTc) are limited.^5^

In 2 studies using the Rice-Vannucci neonatal rat model of unilateral cerebral HI,^6,7^ improved neuropathological and neurofunctional outcomes were observed after an optimized multidose azithromycin regimen, with a loading dose at 2 hours after HI, followed by maintenance doses every 24 hours. Reduction in cerebral infarct size and improved sensorimotor function were observed in both HI injury^6^ and 2 inflammation-amplified HI (IA-HI) models.^7^ Early azithromycin administration (within 1–2 hours of injury) and multidosing covering several days provided the best protection. Neuroprotection was associated with whole blood peak concentration (C_max_) of ≈6 to 10 mg/L and brain tissue levels of 1.5 to 2 mg/kg at 48 hours.^6^ Although mechanisms of protection were not explored in these studies, azithromycin demonstrates immunomodulatory properties in preclinical studies of stroke^8^ and spinal cord injury.^9^ Further safety and validation of efficacy are needed in neonatal large animal studies before early phase clinical trials of azithromycin in HIE.

In this well-characterized model of IA-HI in newborn piglets,^10,11^ the aim was to assess the safety and efficacy of intravenous azithromycin based on neurophysiological (aEEG/EEG background activity), neuroimaging (magnetic resonance spectroscopy [MRS]), and neuropathological (immunohistochemistry) outcomes. Because of known safety concerns in adult humans,^12^ we evaluated whether azithromycin prolonged the QTc interval or caused other adverse effects on cardiorespiratory stability. An IA-HI insult was used to model the perinatal infection and inflammation risk factors associated with HIE in LMICs,^3^ where HT is not protective.^11^ Compared with a pure HI insult in piglets, the IA-HI piglet model is associated with increased morbidity with acute kidney injury, higher mortality, and greater cortical and white matter injury,^10^ with similarities to HIE in LMICs.^13^ We hypothesized that intravenous azithromycin therapy, achieving tissue and plasma concentrations previously shown to be neuroprotective in the Rice-Vannucci rat model,^6^ would be associated with improvement in neurological outcomes in this newborn piglet model.

## Methods

The study was approved by the University College London ethics committee, conducted in accordance with the UK Home Office Regulations [Animals (Scientific Procedures) Act 1986], and adheres to the ARRIVE 2.0 reporting guidance (Animal Research: Reporting of In Vivo Experiments; Supplemental Material).^14^

### Availability of Data

The data that support the findings of this study are available from the corresponding author upon reasonable request.

### Sample Size

In previous piglet studies, we observed a treatment difference of 0.5 to 0.77^15–17^ log_10_ reduction in ^1^H MRS lactate to N-acetyl aspartate peak area ratio (Lac/NAA) with a SD of 0.34 to 0.42.^10,11^ Using a pessimistic prediction of 0.5 Log_10_ unit reduction in basal ganglia and thalamus Lac/NAA and a SD of 0.4, 12 piglets per group were required to achieve 80% power at 5% type I error rate.

### Study Design

**Figure 1.**
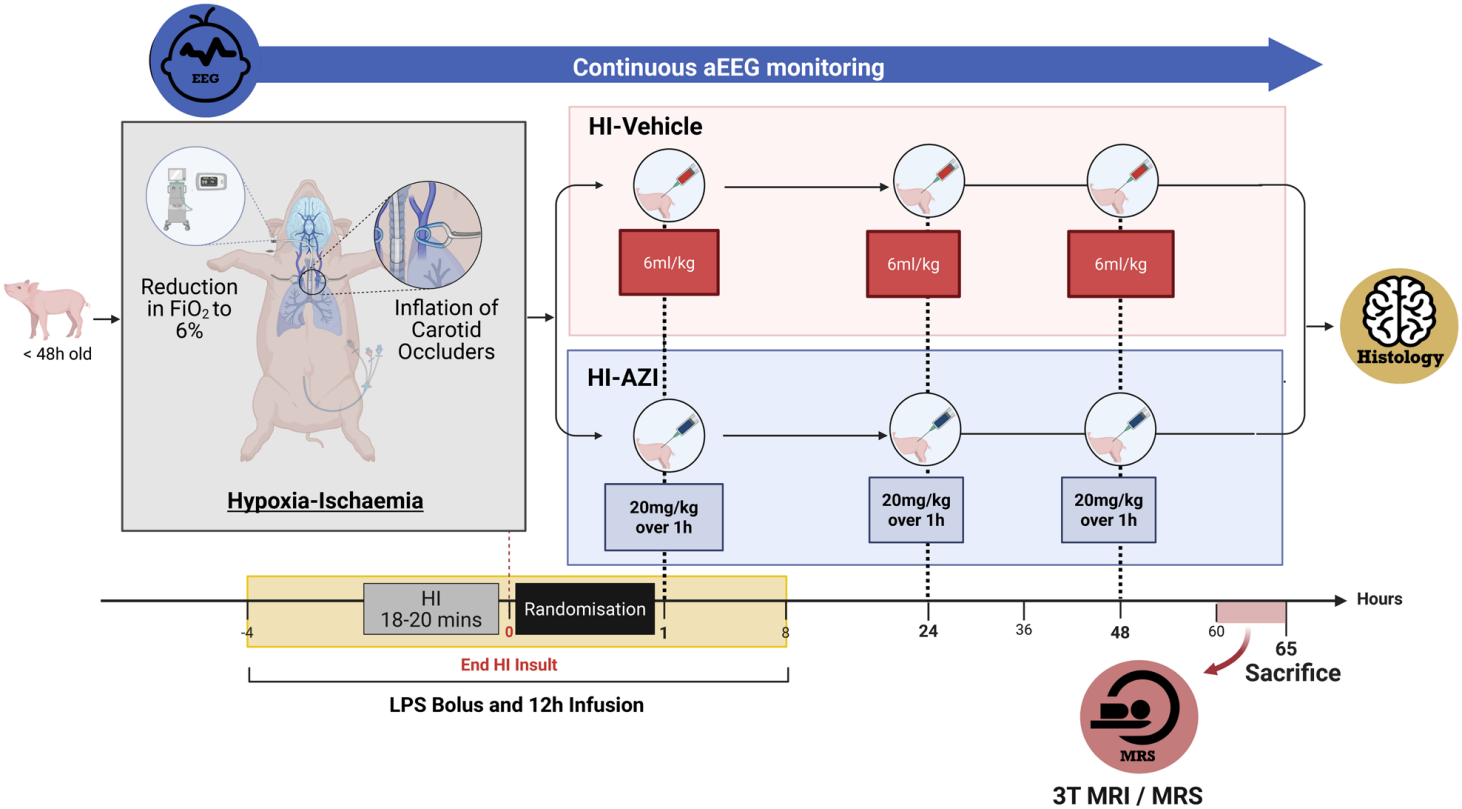
**Experimental protocol.** Newborn large white piglets were examined for good health and surgically prepared, as previously described.^18^ Piglets received an intravenous *Escherichia coli* O157 lipopolysaccharide (LPS) bolus and 12 hours intravenous infusion. At 4 hours into the infusion, piglets underwent an inflammation-amplified hypoxia-ischemia (IA-HI) insult (reduction in FiO_2_ to 6% and transient inflation of bilateral carotid artery occluders). At 1 hour after IA-HI, piglets meeting the eligibility to enter the study were randomized to receive (1) IA-HI–vehicle (n=12) or (2) IA-HI–azithromycin (AZI; n=13). All piglets received full neonatal neurocritical care management for the full duration of the experiments, including continuous amplitude-integrated encephalography (aEEG/EEG) monitoring and 3T magnetic resonance imaging/magnetic resonance spectroscopy (MRS) at 62 to 65 hours. After 65 hours, piglets were euthanised and brain dissected for immunohistochemistry. MRI indicates magnetic resonance imaging. Created in BioRender. Pang, R. (2026) https://BioRender.com/o3xp55b.

Newborn male and female Large White piglets, <48-hour-old, were used in this IA-HI model as previously described.^18^ The experimental protocol is shown in Figure 1. Full details of animal care, surgical preparation and the neurocritical care management are described in the Supplemental Material. Piglets were housed in a purpose-built magnetic resonance imaging safe incubator while receiving full neonatal neurocritical care over 4 days and euthanized at 65 hours post IA-HI with intravenous pentobarbital. Normothermia was maintained throughout at 38 °C using a servo-controlled system (Criticool, Belmont Medical).

### Inflammation-Amplified HI

Piglets underwent inflammation-sensitization with an intravenous bolus of 2 μg/kg *E. coli* lipopolysaccharide O55:B5 (Sigma-Aldrich, Germany) followed by a continuous infusion of 1 μg/kg per hour for 12 hours. An IA-HI insult was performed at 4 hours into the lipopolysaccharide infusion. The carotid artery occluders were inflated, and FiO_2_ was titrated to 6%. The duration of HI was 18 to 20 minutes, determined by blood lactate (target 10–12 mmol/L), duration of isoelectric aEEG/EEG, and duration of hypotension (mean arterial blood pressure 27–30 mm Hg). At the conclusion of HI, occluders were deflated, FiO_2_ was raised to 21%, piglets were resuscitated and randomized using a blinded computer-generated random allocation process at 1 hour after HI to receive either intravenous (1) vehicle (IA-HI–vehicle) or (2) azithromycin (IA-HI–azithromycin).

### Azithromycin Pharmacokinetics Studies

A dose-finding pilot pharmacokinetic study was performed at 2 doses: low-dose (12 mg/kg loading dose over 1 hour+6 mg/kg at 24 and 48 hours) and high-dose (24 mg/kg loading dose over 1 hour+12 mg/kg at 24 and 48 hours; see Figure S1). After the pilot study, intravenous azithromycin (Zedbac, Aspire Pharma, United Kingdom) at 20 mg/kg over 1 hour at 1 hour, 24 hours, and 48 hours after HI was identified as the optimal dosing regimen to achieve neuroprotective concentrations of azithromycin previously reported in rats.^6,7^ The azithromycin was reconstituted in accordance with the manufacturer’s instructions with sterile water for injection, followed by dilution in 48 mL of 0.9% sodium chloride to a final azithromycin concentration of 2 mg/mL. The IA-HI–vehicle animals received an equivalent fluid infusion of 6 mL/kg. A population-pharmacokinetic model for IV azithromycin was fitted as described in the Supplemental Material.

A further pilot study was conducted in 2 animals to optimize neuroprotection with 60 mg/kg administered intravenously over 1 hour at 1 hour, 24 hours, and 48 hours to achieve peak plasma azithromycin levels of ≈8 mg/L based on reassuring safety and pharmacokinetic data from preterm infants receiving intravenous azithromycin at 20 mg/kg.^19^

### Neuromonitoring and Neurological Outcomes

#### Amplitude-Integrated Encephalography

Piglets underwent continuous 8-lead aEEG/EEG (Nicolet, Natus) monitoring throughout the experiment. Electrographic seizures were treated according to standard neonatal guidelines (first line: phenobarbitone, second line: levetiracetam). At the end of the study, the hourly aEEG/EEG background activity was classified independently by 2 lab members blinded to the treatment group using the voltage criteria described by Hellstrom-Westas.^20^ An isoelectric trace was scored 0, and a normal voltage trace was scored 4.

#### Magnetic Resonance Spectroscopy

At 60 hours, ^1^H MRS was acquired at 3T (Philips Achieva, MRI scanner) using chemical shift imaging with an 8×8 matrix in 2 8×8×10 mm^3^ voxels: the basal ganglia and thalamic voxel over the left thalamic region, and the white matter voxel at the level of the centrum semiovale. Spectral acquisition was acquired with a relaxation time of 2000 ms and a long echo time of 288 msec as standard. Final postimaging processing was carried out at the end of the study using TARQUIN, and the Lac/NAA peak area ratio was calculated. The Lac/NAA more accurately represents lactate+threonine/ N-acetylaspartate+N-acetylaspartylglutamate.

#### Immunohistochemistry

At 65 hours, piglets were euthanized with pentobarbital. The brain was perfusion-fixed by intracardiac cold PBS followed by 4% paraformaldehyde. The brain was subsequently dissected, immersed in 4% paraformaldehyde for 1 month, and then embedded in paraffin. For immunohistochemistry, 8 µm sections were cut from 2 coronal slices at the level of the optic chiasm (R0) and hippocampus (R1) from the right hemisphere. Slides were stained and quantified for terminal deoxynucleotidyl transferase-mediated dUTP nick-end labeling (TUNEL+) cell density for cell death, neuronal nuclear antigen (NeuN+) cell density for neuronal survival, Iba1 cell count for myeloid neuroinflammation, OLIG2 (oligodendrocyte transcription factor 2)+ cell density for oligodendrocyte survival, and GFAP (glial fibrillary acidic protein) for astrocytosis in 8 brain regions at x40 magnification (Supplemental Material for detailed methodology). With the exception of the hippocampus, 6 fields of view were assessed (over 2 slices) in all immunohistochemistry analysis. The hippocampus was present in the R1 section only, and therefore, 3 fields were assessed.

The Iba1 ramification index was derived using a 50 µm-by-50 µm sampling grid containing 3 horizontal and 3 vertical lines placed over each field of view at ×40 magnification. This technique reflects the average process complexity per microglial cell within each field. For each field, we counted the total number of Iba1+ process intersection with the gridlines and the number of complete somata within the grid. Fragmented and ambiguous profiles were excluded. The ramification index was defined as follows:


$$Ramif\;ication\;\;\;Index=\;\frac{{\left(Number\;\;\;of\;\;\;branch\;\;\;intersections\right)}^{2}}{Number\;\;\;of\;\;\;intact\;\;\;Iba1+somata}$$

To confirm that findings were consistent at higher resolution, a supplementary validation analysis was performed at ×60 magnification.

### Biochemical Assays

Plasma and brain tissue azithromycin levels were measured using liquid chromatograph–tandem mass spectrometry, as previously described.^6^ An ex vivo endotoxin-stimulated cytokine study was performed using 0.5 mL of whole blood stored in lithium heparin blood tubes, spiked with 10 ng/mL of *E. coli* lipopolysaccharide for 4 hours at 37 °C agitated at 100 rpm, paired against an unstimulated blood sample spiked with 5 µL of PBS.^21^ All blood samples were centrifuged at 3000 relative centrifugal force, and plasma was stored at −80 °C until batch analysis at the end of the study. Plasma cytokines (IL-1ra, IL [interleukin]-4, IL-6, IL-10) levels were measured using a porcine-specific, Luminex Discovery Multianalytes Assay (Bio-Techne, R&D Systems, Minneapolis) following the manufacturer’s instructions. Plasma TNF (tumor necrosis factor) α was measured using the human Simple-Plex platform (Bio-techne, R&D Systems, Minneapolis) in accordance with the manufacturer’s instructions. Full blood count was measured by the Clinical Pathology Laboratory at the Royal Veterinary College (Hertfordshire, United Kingdom). The systemic inflammation response index was deduced using neutrophils×monocytes/lymphocytes.

### Statistical Analysis

Statistical analysis was performed using JMP (v17, SAS), GraphPad Prism (v9), and R-studio (v.4.0.5). All physiological data, biochemical, and hematological results were assessed using an ANOVA model with fixed factor effects of treatment, time, and treatment×time interaction plus a random effect subject to account for repeated measures where necessary as previously described.^16^ Treatment groups were compared using 95% CI for the difference in the least square means, and *P* values were deduced.

For neurological outcome data (MRS, aEEG/EEG, and immunohistochemistry), a Bayesian statistical approach was used (Cytel, Cambridge, MA) to determine the probability of treatment superiority (Pr_[__sup]_) using noninformative priors. This approach avoids dichotomization of data and enables direct hypothesis testing (for treatment benefit), therefore deducing inferences that are more intuitive and meaningful. For comparison, frequentist analyses of selected outcome data were also performed and presented in the Supplemental Material. Simulations were conducted under alternative effect sizes to determine the decision rules meeting the 2.5% 1-sided type I error rate with adequate power (>80%) for early stopping of the study. For treatment futility, the posterior probability of treatment superiority (Pr_[__sup]_) of 50% or less was associated with the predicted probability of early stopping under the null hypothesis of 49.8% with 91.6% power and 5% error rate. MRS Lac/NAA data were Log_10_ transformed as standard practice. Bayesian linear regression with noninformative priors for the entire sample as well as the 2 sex groups was fitted to estimate the treatment effect. The posterior probability in basal ganglia and thalamus and white matter Lac/NAA (azithromycin versus saline), treatment effect estimates, and 95% credible intervals (CrI) were calculated using Markov Chain Monte Carlo sampling. For aEEG/EEG, the background hourly aEEG/EEG scores were aggregated in 6-hourly intervals. A Bayesian linear mixed effects model with a power transformation of the aggregated encephalography scores was fitted, and treatment effects were estimated. The probability of superiority and the 95% CrI were calculated using the same method as the MRS outcomes. For immunohistochemistry outcomes, a multioutcome multilinear model was used, adjusting for sex, fitted to estimate the treatment effect on histological counts at the regional level. Multiplicity adjustment of treatment effect *P* values was performed to control the false discovery rate using the Benjamini-Hochberg method. Missing values for each of the 8 regions were imputed using multiple imputation by chain equations. Sex-stratified analyses were conducted for the MRS and aEEG outcomes; however, as the study was not powered to detect sex-specific effects, these analyses were exploratory and are reported descriptively.

## Results

### Baseline Characteristics

Four piglets were excluded: 2 animals were euthanized after surgery before lipopolysaccharide sensitization due to poor health, 1 IA-HI-vehicle animal died at 50 hours after IA-HI, before MRS acquisition, due to refractory hyperkalemia secondary to severe renal failure, and 1 IA-HI–azithromycin animal was excluded after HI as the injury was too severe, falling outside the standard insult parameter range. Twenty-five animals were studied and randomized to either (1) IA-HI–vehicle (n=12) or (2) IA-HI–azithromycin (n=13). Physiological and biochemical parameters for the IA-HI insult were similar across the groups (*P*>0.05; see Table 1).

**Table 1.**
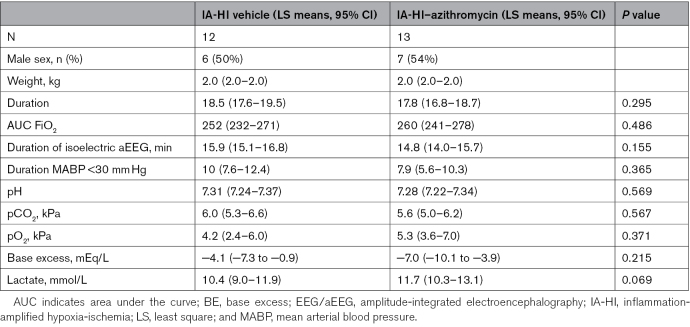
Baseline and Insult Characteristics

### Physiological Response

We observed no overall intergroup differences in the physiological (rectal temperature, heart rate) or biochemical blood gas (pH, pCO_2_, Base Excess, Lactate, Glucose) parameters throughout the duration of the experiments (Table S1). The creatinine increased with time (*P*<0.001) and was lower in IA-HI–azithromycin versus IA-HI–vehicle at 48 to 65 hours (least square mean difference 68µmol/L [95% CI, 15–120]; *P*=0.01). Across the groups, 10 of 25 (40%) animals developed acute kidney injury requiring treatment for hyperkalemia (vehicle n=7, azithromycin n=3).

**Figure 2.**
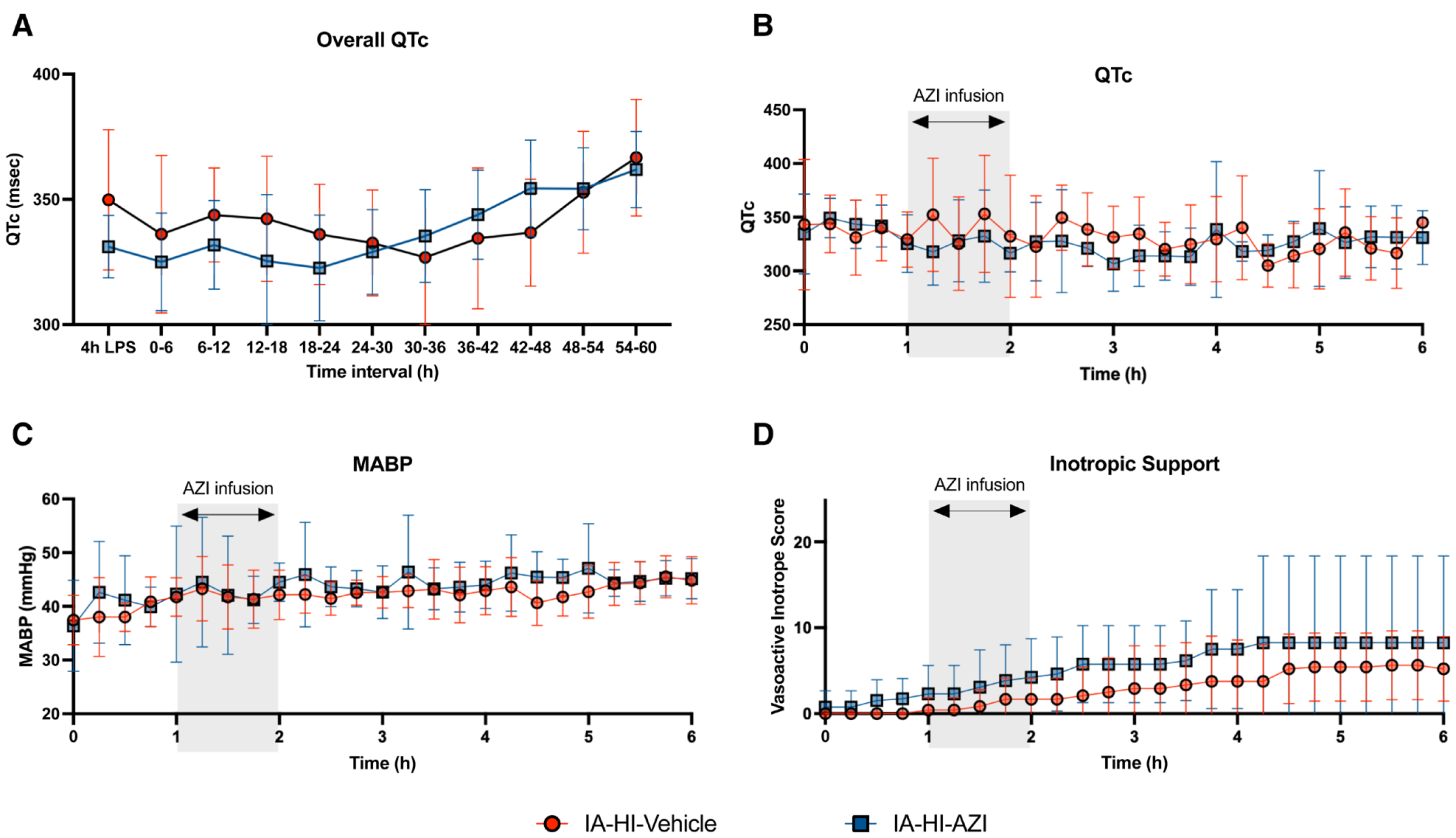
**Hemodynamic effect of azithromycin (AZI) infusion.** Inflammation-amplified hypoxia-ischemia (IA-HI)–AZI 20 mg/kg was not associated with significant prolongation in the correct QT interval (QTc) over the full duration of experiments (**A**) or during azithromycin infusion (**B**) compared with IA-HI–vehicle. We also observed no difference in mean arterial blood pressure (MABP; **C**) or inotropic support requirements (**D**).

The mean arterial blood pressure and vasoactive inotropic score were not significantly different between the groups. We observed no significant differences in QTc values during azithromycin infusion and throughout the entire duration of the experiments compared with vehicle (*P*>0.05), as shown in Figure 2.

### Pharmacokinetics

Intravenous azithromycin at 20 mg/kg over 1 hour given at 1 hour after IA-HI, repeated at 24 and 48 hours, was associated with C_max_ of 1.89±0.65, 1.90±0.74, and 1.97±0.48 mg/L at 2, 25, and 49 hours after IA-HI, respectively (Figure S2). Mean brain azithromycin levels were 1.45±0.45 mg/kg from 5 animals.

The pharmacokinetic parameter estimates derived from a population-based pharmacokinetic model are shown in Table 2. A 2-compartmental model with proportional error provided the best fit, demonstrated by the goodness of fit plots (Figure S2) and reduction in Akaike Information Criterion. Based on an average piglet weight of 2 kg, the half-life of azithromycin was 33 hours and the area under the curve ∝ of 12.3 mg×h/L.

**Table 2.**
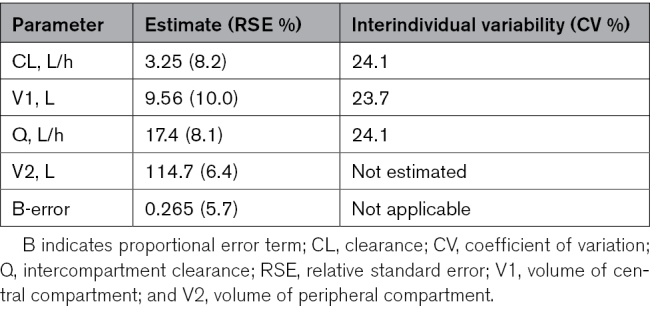
Population-Based Pharmacokinetic Parameter Estimates for Azithromycin

### Magnetic Resonance Spectroscopy

**Figure 3.**
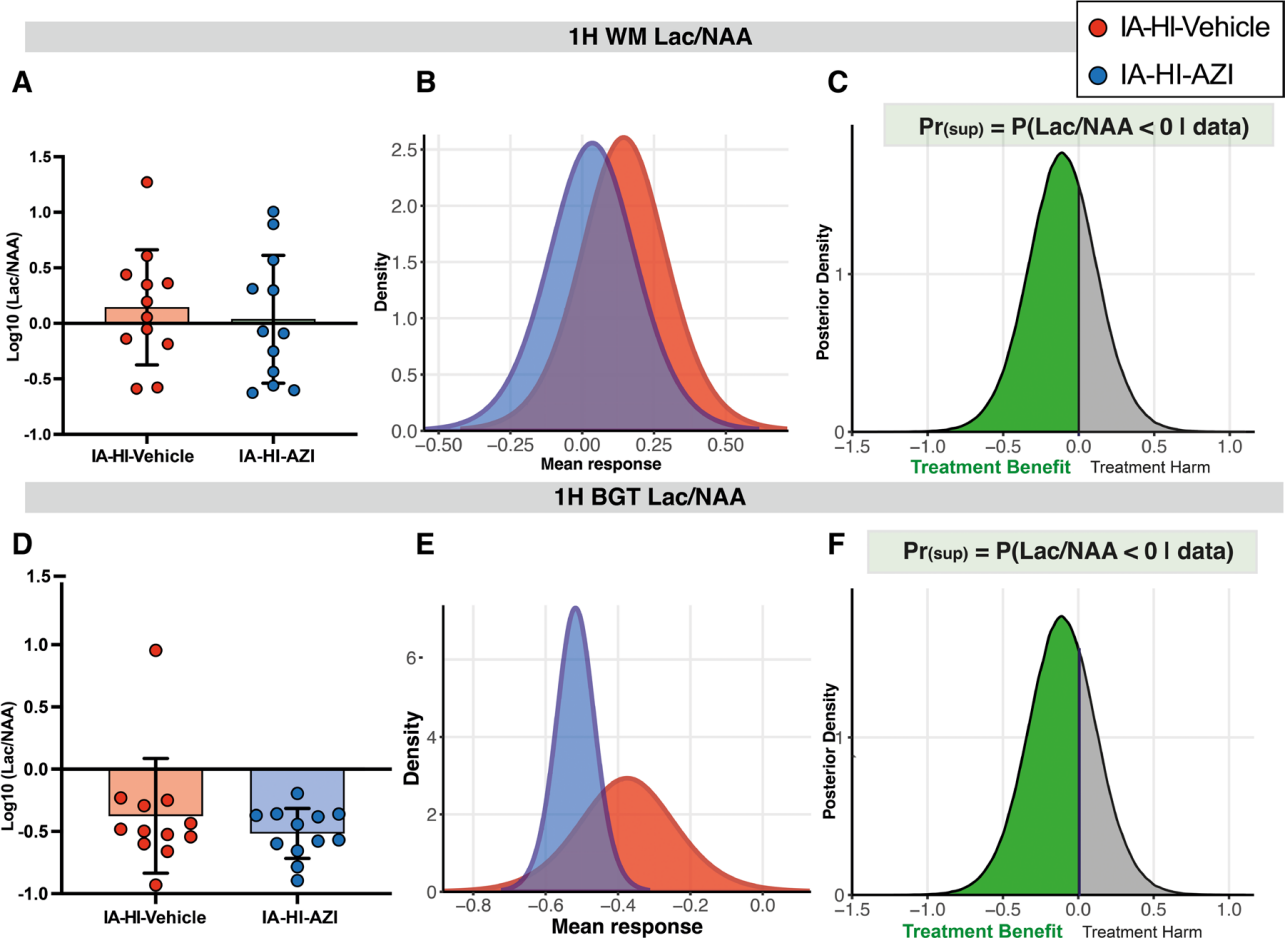
**Magnetic resonance spectroscopy (MRS).** ^1^H MRS lactate to N-acetyl aspartate peak area ratio (Lac/NAA) peak area ratio between inflammation-amplified hypoxia-ischemia (IA-HI)–azithromycin (AZI) vs IA-HI–vehicle in the white matter (WM) voxel (**A** through **C**) and basal ganglia and thalamus (BGT) voxel (**D** through **F**). Data presented as metabolic ratios in individual piglets (dots) with group mean (bar) ±SD in **A** and **D**, density plots of the distribution of the grouped data in **B** and **E**. The posterior distribution plots that follow Bayesian analysis with noninformative priors are shown in **C** and **F**. The probability of superiority (Pr_[sup]_) in azithromycin to reduce Lac/NAA was 68.5% and 82.7% in the WM and BGT voxels, respectively.

^1^H MRS Lac/NAA data were available for 24 animals (vehicle n=12 and azithromycin n=12) and shown in Figure 3. The magnetic resonance imaging scanner was not available for 1 animal in the IA-HI–azithromycin group.

The Pr_(sup)_ to reduce the Lac/NAA peak area ratio with IA-HI–azithromycin was 82.7% in the basal ganglia and thalamus voxel (log_10_ unit difference, −0.143 [95% CrI, −0.456 to 0.170]) and 68.5% in the white matter voxel (−0.109 [95% CrI, −0.576 to 0.356]). The sex-stratified analysis showed inconsistency between groups with wide CrIs (Table S2).

### Electroencephalography

**Figure 4.**
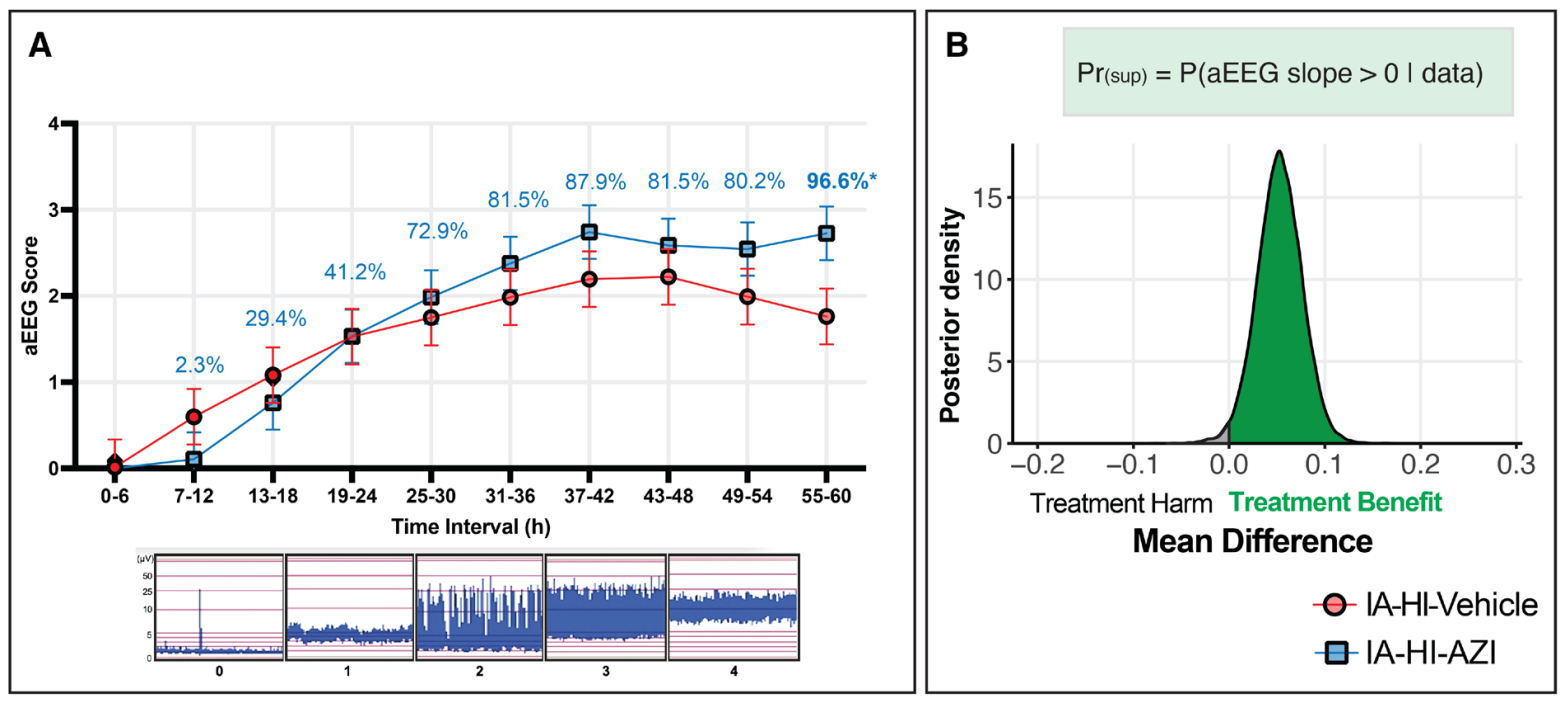
**Amplitude-integrated encephalography (aEEG/EEG).** aEEG/EEG were classified in accordance with the voltage criteria every 1 hour and averaged over 6 hours, with the probability of superiority (Pr_[sup]_) shown for each time epoch. A Bayesian linear mixed effects (LME) model was applied to the hourly scores and Pr_(sup)_ deduced on the entirety of the data. The Pr_(sup)_ for azithromycin (AZI) to improve background aEEG activity over 60 hours after inflammation-amplified hypoxia-ischemia (IA-HI) was 98.6%.

Background aEEG/EEG data were available for all 25 piglets and shown in Figure 4. Four animals (16%) developed seizures; the incidence did not differ between groups (vehicle n=2, azithromycin n=2).

Azithromycin was associated with significant improvement in background aEEG/EEG recovery overall with Pr_(sup)_ of 98.6% (treatment difference, 0.05 [95% CrI, 0.006–0.09]; see Figure 4B). At 55 to 60 hours, azithromycin was associated with significant improvement in aEEG/EEG score (mean difference 0.461 [95% CrI, −0.040 to 0.961], Pr_[__sup]_=96.6%; Figure 4A). The sex-stratified descriptive data are shown in Table S2.

### Immunohistochemistry

**Figure 5.**
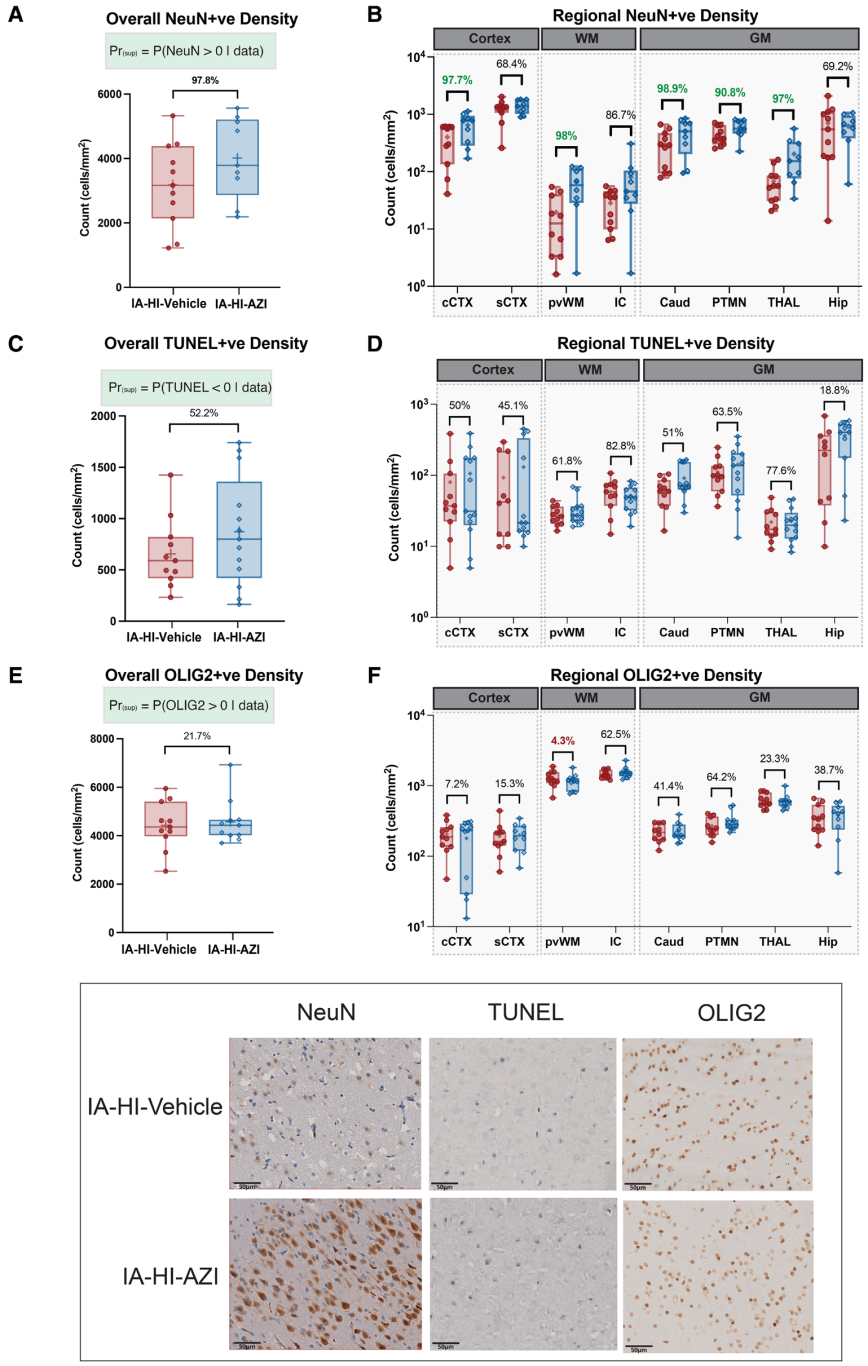
**Immunohistochemistry.** Eight brain regions were assessed for neuronal nuclear antigen (NeuN)-positive cell density (**A** and **B**), terminal deoxynucleotidyl transferase mediated dUTP nick end labeling assay (TUNEL)-positive density (**C** and **D**), and OLIG2 (oligodendrocyte transcription factor 2)–positive cell density (**E** and **F**). Data shown as overall (**A**, **C**, and **E**) and regional means±SEM (**B, D**, and **F**). The probability of treatment superiority is shown where Pr>94.8% (superiority threshold), highlighted in green. Representative micrographs from the cingulate cortex (NeuN), caudate (TUNEL), and internal capsule (OLIG2) are shown. AZI indicates azithromycin; CAUD, caudate nucleus; cCTX, cingulate gyrus; GM, gray matter; Hip, hippocampus; IA-HI, inflammation-amplified hypoxia-ischemia; IC, internal capsule; Pr_(sup)_, probability of superiority; PTMN, putamen; PvWM, periventricular white matter; sCTX, sensorimotor cortex; THAL, thalamus; and WM, white matter.

Brain immunohistochemistry was assessed for neuronal cell density (NeuN [cells/mm^2^]), TUNEL-positive cell death (cells/mm^2^), microglia cell density (Iba1 [ionized calcium-binding adapter molecule 1]+cells/mm^2^), activation status (ramification index), and oligodendrocyte cell density (OLIG2+ cells/mm^2^) for all animals (n=25). The data are shown in Figure 5.

IA-HI–azithromycin was associated with an overall increase in NeuN-positive cells compared with IA-HI-vehicle, with a Pr_(sup)_ of 97.8% (mean difference 95% CrI, 0.01–0.72). We observed a significant increase in NeuN+ cell density in the cingulate cortex, periventricular white matter, caudate, and thalamus (Pr_[__sup]_ 97.7%, 98.0%, 98.9%, 99.7% and 97%, respectively).

TUNEL+ cell count and OLIG2+ cell density were not significantly different between groups. The overall probability of azithromycin reducing TUNEL-positive cell count and increasing OLIG2-positive cells was 52.2% and 41.8%, respectively.

### Neuroinflammation

#### Microglia Activation State

The overall posterior probability of increased Iba1+ cell count with IA-HI–azithromycin was 99.6%. Regional analysis demonstrated significantly higher counts within the cingulate cortex, periventricular white matter, caudate, putamen, and thalamus (posterior probability 99.9%, 94.7%, 100%, 100%, 93.4%).

The activation state of the microglia was also assessed using a ramification index. Resting microglia are highly ramified, reflected by a high ramification index but become ameboid in shape with fewer cell processes upon activation, reflected by a reduction in the ramification index. IA-HI–azithromycin was associated with a significant increase in the ramification index, suggesting a resting morphology of the microglia. Overall, the probability for higher Iba1 ramification index was 99.6% in IA-HI–azithromycin compared with IA-HI-vehicle (difference, 0.17 [95% CrI, 0.05–0.30]). Regional analysis demonstrated significantly higher ramification index in the periventricular white matter, internal capsule, caudate, thalamus, and putamen with the Pr_(sup)_ of 94.7%, 96.7%, 99.5%, 99.5%, 92.2% respectively. A ×60 high-magnification sensitivity analysis yielded a consistent overall treatment effect (Table S3).

#### Systemic Blood Biomarkers of Inflammation

We observed no difference in total white cell, neutrophil, and lymphocyte counts between groups over time (*P*>0.05; see Figure S3). IA-HI–azithromycin was associated with higher platelet count at 65 hours (least square mean difference, 92.6 [95% CI, 25.8–159.5]; *P*=0.007) and a lower systemic inflammatory response index at 24 hours after azithromycin administration (least square mean difference, 0.48 [95% CI, 0.1–0.86]; *P*=0.013).

**Figure 6.**
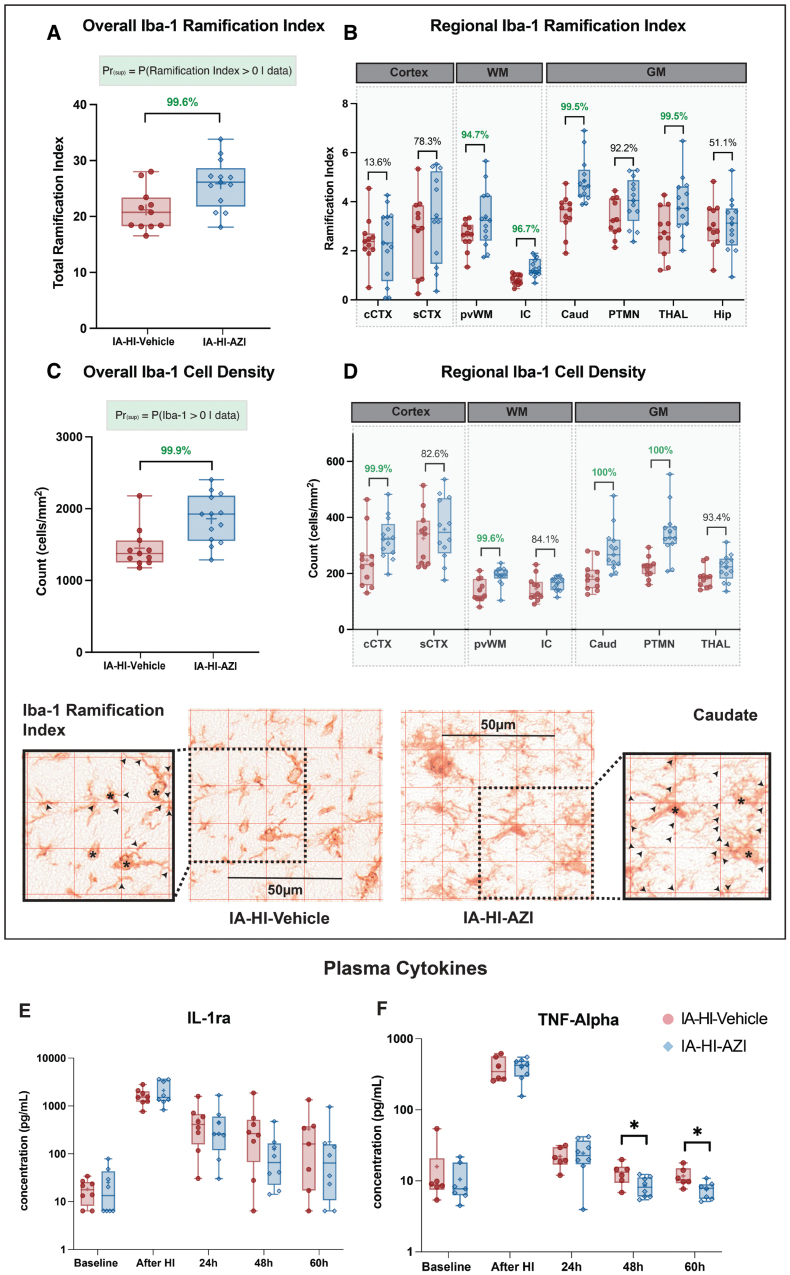
**Inflammatory response.** To assess the immunomodulatory effects of azithromycin, microglia (Iba1 [ionized calcium-binding adapter molecule 1]+ cells) morphology (**A** and **B**) and cell density (**C** and **D**) were assessed in 8 regions of the brain on immunohistochemistry. Data shown as overall (**A** and **C**) and regional means±SEM (**B** and **D**). The probability of treatment superiority is shown, where Pr>94.8% (superiority threshold), highlighted in green. Representative micrographs acquired at ×40 magnification are shown; asterisks denote microglial cell bodies, and arrowheads indicate processes included in the ramification analysis. To assess the systemic inflammatory response, interleukin (IL)-1ra (**E**) and TNF (tumor necrosis factor) α (**F**) were measured at baseline, immediately after inflammation-amplified hypoxia-ischemia (IA-HI), and at 24, 48, and 60 hours. Applying an ANOVA model with log_10_-transformed data showed group differences in TNFα (square bracket where *P<0.05) at 48 and 60 hours. AZI indicates azithromycin; CAUD, caudate nucleus; cCTX, cingulate gyrus; GM, gray matter; Hip, hippocampus; IC, internal capsule; Pr_(sup)_, probability of superiority; PTMN, putamen; PvWM, periventricular white matter; sCTX, sensorimotor cortex; THAL, thalamus; and WM, white matter

Plasma TNFα, IL-1ra, IL-4, IL-6, IL-10, and IL-12 were measured at baseline, after IA-HI, and at 24, 48, and 60 hours in 14 animals (n=6 in IA-HI–vehicle, n=8 in IA-HI–azithromycin). Plasma cytokines peaked immediately after IA-HI and were below levels of detection in IL-4, IL-6, IL-10, and IL-12 at 24, 48, and 65 hours (Figure S4). Although no group differences were observed in plasma IL-4, IL-6, IL-10, IL-12 (Figure S4), and IL-1ra (Figure 6E), we observed significant reduction in TNFα at 48 hours (log_10_ difference 0.18 [95% CI, 0.02–0.34]; *P*=0.032) and 60 hours (difference, 0.20 [95% CI, 0.04–0.34]; *P*=0.018) in HI-azithromycin compared with IA-HI-vehicle (Figure 6F).

### High-Dose (60 mg/kg) Pilot Study

The pharmacokinetic profile of the further pilot study using 60 mg/kg of azithromycin is shown in Figure S5. Peak plasma azithromycin levels (≈7–10 mg/L) were close to the target range based on rat HI studies^6^; however, safety concerns arose at this higher dose. In both piglets, hypotension required increased inotropic support within 30 minutes of starting the azithromycin infusion (Figure S5B and S5C). Seizures were noted on aEEG/EEG in both cases (from 12 to 21 hours after IA-HI and 79–65 hours after IA-HI, respectively). There were no differences in the QTc interval in azithromycin-treated animals compared with vehicle. Mean brain tissue azithromycin concentration from the 2 piglets was 6.6 mg/L at 65 hours, which was 3-fold higher than in the rat model at a similar time point.^6^

## Discussion

In this IA-HI piglet model relevant to the LMIC settings, intravenous azithromycin at a dose of 20 mg/kg administered 1 hour after IA-HI and repeated at 24 and 48 hours was safe and beneficial based on neurophysiological and neuropathological outcomes. The treatment benefit manifested as a late recovery in aEEG/EEG activity, improvement in neuronal cell density (NeuN), and a reduction in microglial activation, with the probability of treatment superiority of 98.6%, 97.8%, and 99.6%, respectively. The treatment effects on MRS Lac/NAA and TUNEL-positive cell death were modest, suggesting a predominant immunomodulatory role of azithromycin, which may complement other therapies previously investigated, such as melatonin.^18^

We observed no treatment-related adverse events associated with intravenous azithromycin administration based on physiological and biochemical parameters at 20 mg/kg intravenously. In particular, QTc was not significantly prolonged compared with IA-HI–vehicle. The mechanism of QTc prolongation with macrolide therapy relates to inhibition of cardiac hERG (human ether-à-go-go-related gene) potassium channels, leading to delayed repolarisation.^22^ The mRNA expression of common cardiac ion channels is similar between humans and pigs, with comparable repolarization currents, and rate- and temperature-dependent responses, supporting the utility of this species for cardiac safety research.^23^ Animal studies suggest azithromycin is not proarrhythmogenic,^24^ and no evidence of QTc prolongation have been observed in clinical studies of children who received azithromycin^25,26^ although data in neonates are limited.^5^ While azithromycin at 20 mg/kg is regarded as safe in the preterm neonatal population,^5^ further safety studies in early phase trials are needed in infant with HIE undergoing HT, which is also known to increase the QTc.^27^

Intravenous azithromycin 20 mg/kg over 1 hour achieved plasma C_max_ levels of ≈1.9 mg/L and brain tissue azithromycin levels of ≈1.5 mg/L; these levels were below the putative neuroprotective target plasma levels of 2 to 3 mg/L, and 25% to 50% lower than the brain tissue levels of 2-3 mg/kg reported in rats by Barks et al^6^ We explored the safety of increasing the dose (aiming for C_max_ of 8 mg/L based on existing safety data in preterm infants^19^); however, we observed toxicity, with hypotension and seizures at 60 mg/kg (plasma levels 7–10 mg/L and brain levels 6.6 mg/L)in a small pilot study. This toxicity of high-dose azithromycin aligns with data in adult micro-pigs where azithromycin plasma levels of ≈120 mg/L, were associated with dose-dependent hypotension, atrioventricular conduction block, and cardiac arrest within 30 minutes of infusion.^28^ Such levels were ≈10-fold higher than in our newborn piglet study, suggesting increased vulnerability of the immature heart, which may have been further exacerbated by the systemic inflammatory and hypoxic insult. When translating azithromycin to early phase trials, it is important to consider existing data reporting significantly reduced drug clearance, including intercompartmental clearance, which may predispose neonates to greater drug accumulation and tissue exposure.^19^ The prolonged half-life in human neonates (69 hours,^19^ ≈3-fold higher than piglets) may further enhance drug penetration across the blood-brain barrier, increasing brain exposure and potential risk of neurotoxicity. These findings highlight the need for careful dose optimization and close monitoring to ensure safety in this vulnerable population.

These piglet data demonstrate that azithromycin at 20 mg/kg is associated with widespread improvement in neuronal density across multiple brain regions, together with improved recovery in aEEG/EEG background activity, particularly notable from ≈55 hours after IA-HI. Our data suggest that azithromycin modulates the microglial response to IA-HI injury; we observed an increase in number of Iba1-positive microglia with azithromycin, which exhibited a more resting and ramified morphology compared with the vehicle group. Microglia play critical roles in both the injurious and reparative processes after HI.^29^ Photon imaging in vivo shows resting, ramified microglia as highly dynamic cells; whereas their cell bodies remain largely static, their processes extend and retract continuously, surveying the parenchyma once every few hours. This motility facilitates immune surveillance, shields injured sites, and supports phagocytic clearance of injured cells and cellular debris.^30^ Beyond immunosurveillance, microglia respond to neuronal distress signals and directly interact with neurons to modulate network activity.^31^ Disease states such as stroke can shift microglia toward a maladaptive phenotype, which reduces neuroblast survival and impacts negatively on neurogenic repair.^32^ The observed increase in Iba1+ microglia numbers with a resting phenotype after azithromycin treatment may represent improved microglia survival and a shift toward supporting homeostasis. Importantly, this ramified microglial state coincided with improved NeuN-positive neuronal density and associated aEEG/EEG recovery, which we speculate may contribute to preservation of neuronal network stability and a reduction in excitotoxic injury.

In preclinical stroke and spinal cord injury models, azithromycin promotes microglial and macrophage polarization toward an anti-inflammatory M2-like phenotype^8,9,33,34^ via JAK-STAT signaling^35^ and suppression of NFκB (nuclear factor kappa B) activation.^34^ In vitro, azithromycin inhibits lipopolysaccharide-induced NFκB/p65 translocation to the nucleus in microglia, which coincides with a reduction in IL-1β, TNFα, and IL-6 secretion.^36^ Although the debate over the validity of the M1/M2 paradigm remains, our data support the presence of an anti-inflammatory shift with azithromycin. In addition to the morphological differences in microglia with azithromycin treatment, we also observed a significant reduction in plasma TNFα. Plasma measurements reflect systemic changes and may underestimate the local tissue-level inflammatory response, but this is an important finding given a recent report of the negative impact of TNF-driven influences on neural stem and progenitor cell differentiation in stroke.^32^ Together with in vitro data showing the influence of TNFα^37^ and other cytokines^38^ on neural precursor activity, our findings support azithromycin’s immunomodulatory role in supporting an environment for neuronal survival and repair, which aligns with our observation of improved NeuN density in azithromycin-treated animals.

There were some unexpected findings in this study. Although azithromycin-treated groups showed increased NeuN+ and Iba1 density, TUNEL-positive cell counts remained similar compared with vehicle. This contrasts with the pattern of neuroprotection seen with melatonin^18^ where TUNEL-positive cell counts were reduced and suggests that different therapies may shift cell death to different extents along the apoptosis–necrosis neuronal cell death continuum.^39^ Another unexpected result was the observation of a trend to reduced OLIG2+ cell density in azithromycin-treated groups, suggesting a possible disruption in myelination. These findings contrast with the benefit observed in oligodendrogenesis and oligodendrocyte maturation seen in in vivo^40^ and in vitro^36^ studies of azithromycin, with associated improvement in neuromotor outcomes.^6,7^ In the ovine model, azithromycin increased mature oligodendrocyte density and myelin volume, despite no change in OLIG2+ staining.^40^ These findings concur with in vitro data demonstrating azithromycin’s ability to ameliorate developmental arrest in oligodendrocyte progenitor cells after lipopolysaccharide-induced microglial injury, while promoting the maturation of APC (activated protein C)^+^ oligodendrocytes and increasing myelination.^36^ Similar mechanisms of neuroprotection have been observed in other agents such as erythropoietin^41–43^ and insulin-like growth factor-1.^44–46^

Our study supports the safety and benefit of azithromycin; however, it contrasts with the modest neurological benefit reported in an ovine model of umbilical cord occlusion. Mike et al^40^ administered intravenous azithromycin to pregnant ewes 1 hour before umbilical cord occlusion followed by 3 intravenous azithromycin doses of 15 mg/kg at 1, 24, and 48 hours after HI. Although improvement in white matter immunohistochemistry markers was observed, there was no significant increase in NeuN density or reduction in caspase-3 positive cells within the gray matter. However, the authors reported an improved ability to feed in the azithromycin group, likely reflecting improved alertness at the early stage of recovery. The reason for differences in azithromycin outcomes between the piglet IA-HI model and the ovine model is unclear, despite comparable brain tissue concentrations and higher plasma C_max_ compared with our piglet study. A possible explanation may be the differences in azithromycin preparations, as variations in drug stereochemistry across manufacturers may impact its drug action and therapeutic potential.^47^ In addition, the small sample size and absence of robust outcome biomarkers such as aEEG/EEG and MRS may have limited detection of treatment effects in the ovine study. Finally, there might be maturational stage differences between neonatal sheep and piglets. It is possible that large animal models with early read-outs may underrepresent the therapeutic effects that emerge later in the rodent studies. Our data in piglets aligns with rodent studies by Barks et al,^6,7^ which demonstrate promising functional and neuropathological outcomes at later time points of postnatal day 21 to 35. Importantly, while both our study and the ovine model^40^ demonstrate safety at comparable brain tissue concentrations of azithromycin, these were subtherapeutic in comparison to the brain tissue concentration in rodents,^7^ indicating that further dose optimization may be necessary.

Combination therapies may be needed for optimal benefit in this model of IA-HI, relevant to LMIC settings. Indeed, the benefits of azithromycin may complement therapies such as melatonin, which targets different levels of the neurotoxic cascade, such as scavenging oxygen-free radicals and ameliorating mitochondrial dysfunction.^48^ Rigorous and detailed large animal studies, which study safety, efficacy, and pharmacokinetic will be critical to establish optimal combination treatment strategies before translation to clinical trials.

A strength of this study is the use of the IA-HI model, which was developed to assess therapies for the LMICs, to account for the contribution of infection and inflammation observed in babies with HIE in sub-Saharan Africa and the lack of benefit from HT.^3^ We used a Bayesian approach, which enables explicit quantification of uncertainty^18^ and is well suited to preclinical studies, aimed to assess whether an intervention merits further investigation. For transparency, conventional frequentist analyses were also performed for selected outcomes and showed concordant results (Table S4). In particular, where *P* values did not reach significance, the Bayesian probabilities of superiority still suggested a potential treatment benefit, indicating that the findings warrant careful consideration and support the rationale for further studies to optimize dosing and treatment regimens. There are some limitations of the study. The early read-out of Lac/NAA at 60 hours may not capture the full extent of azithromycin targeting later stages of the neurotoxic cascade. The current piglet model is limited to 65 hours^49^; however, we use surrogate outcome measures, including Lac/NAA, aEEG/EEG, and immunohistochemistry. The study was not powered to examine sex-specific effects, and although small sex-stratified differences were observed in some exploratory analyses, these estimates were highly uncertain and are likely to reflect sampling variability rather than true biological sex dependence.

Azithromycin is an attractive therapeutic intervention given its established safety profile in vulnerable preterm neonates^5^ and benefit in LMICs.^4^ Although neonatal trials have not shown benefit in reducing bronchopulmonary dysplasia,^50^ the safety data at doses comparable to this piglet study (20 mg/kg in the first 3 days) are reassuring.^51^ Large trials in LMICs, including the MORDOR study,^4^ demonstrate mortality benefit in young infants, however, more recent studies of intrapartum^52^ or neonatal prophylactic azithromycin^53^ administration did not reduce mortality. Multidrug resistance remains a key concern when considering the repurposing of azithromycin,^54^ but the duration of therapy would be short for HIE neuroprotection.

In summary, in this IA-HI piglet model of HIE, in which HT is not protective, 20 mg/kg intravenous azithromycin administration within 1 hour of birth and repeated every 24 hours for 2 further doses at 24 and 48 hours was safe and beneficial. We observed no change in any physiological parameter, including QTc. We observed improvement in aEEG/EEG activity from 55 hours, coinciding with significant increases in neuronal cell density and anti-inflammatory effects on brain immunohistochemistry, although there was no reduction in the cerebral MRS Lac/NAA peak area ratio, our main outcome biomarker. While azithromycin appears safe and demonstrates biological benefit at doses of 20 mg/kg per 24 hours, the lack of benefit on TUNEL and OLIG2+ cell density warrants further investigation in future dose optimization studies. Assessment of azithromycin in combination with other agents, such as melatonin, with complementary protective mechanisms, may also hold promise. Further preclinical safety and efficacy studies of azithromycin with HT in HI-only models are needed before translation to settings where HT is routine.

## ARTICLE INFORMATION

Presented in part at the 15th International Newborn Brain Conference, Cork, Ireland, February 28–March 2, 2024, and the Pediatric Academic Societies Meeting, Toronto, Canada, May 2–6, 2024.

### Acknowledgments

The authors thank Drs Debbie Kraus (Prism, Cambridge, United Kingdom), Gerald Smith, and Jiaxuan Wang (Cytel, MA) for their statistical support.

### Sources of Funding

### Disclosures

None.

### Supplemental Material

Supplemental Methods

Tables S1–S4

Figures S1–S5

Major Resources Table

ARRIVE Checklist

## Supplementary Material


