# Supplementary material for: Postnatal Azithromycin Is Neuroprotective and Anti-Inflammatory in a Piglet Model of Hypoxic-Ischemic Encephalopathy
Source: Stroke. 2026 Feb 13;57(5):1362–75. doi: 10.1161/STROKEAHA.125.054318 (PMC13117526; doi:10.1161/STROKEAHA.125.054318)

SUPPLEMENTAL MATERIAL

1 Supplemental Methods

1.1 Animal Care, Surgical Preparation and Neurocritical Care Management

Newborn piglets < 48h of age were examined on arrival for general health. Animals were sedated with an intramuscular injection of 50μg/mL of midazolam followed by induction of anaesthesia using 2-3% of inhaled isoflurane. A midline 2-3cm incision was performed superior to the laryngeal prominence to allow the trachea and both carotid arteries to be isolated by blunt dissection. Piglets were intubated via a tracheostomy using a size 3.5-4mm shouldered endotracheal tube (Smiths Medical, UK) and mechanically ventilated for the remainder of the experiment. Deflated vascular occluders (OC2A, In Vivo Metric, USA) were secured around the common carotid arteries bilaterally. An umbilical venous (4Fr double-lumen catheter, Vygon, Surrey, UK) and peripherally inserted central venous catheters (double-lumen long lines, Vygon, Surrey, UK) in the cephalic or axillary veins were used for infusions. An umbilical arterial catheter (4Fr double-lumen catheter, Vygon, Surrey, UK) was placed for blood pressure monitoring and blood sampling. Following surgery, piglets were transferred to a purpose-built MRI-compatible incubator (LMT Medical Systems, Germany) for continued intensive care.

Full neonatal intensive care management was provided throughout the study duration. Piglets remained anaesthetized and sedated throughout using inhaled isoflurane mixed with air (1.5 – 2.5% v/v) and intravenous fentanyl (3-5 μg/kg/hr). Mechanical ventilation (SLE 2000 infant ventilator, SLE Limited, South Croydon, UK) was adjusted according to arterial blood gases (targeting PaO2 8-13 kPa and pCO2 4.5 – 6.5 kPa). Maintenance intravenous 10% glucose was given at a rate of 60ml/kg/day initially and reduced to 40ml/kg/day post-HI. All animals received intravenous antibiotics (benzylpenicillin, 50mg/kg BD and gentamicin, 5mg/kg OD). Complications of HI, including seizures, hypotension and hyperkalaemia, were treated in line with local neonatal guidelines.

1.2 Histology Methodology

Piglets were euthanised at 65h with intravenous pentobarbital followed immediately by intra-cardiac cold PBS perfusion. The brain was dissected, fixed in 4% paraformaldehyde (PFA) and embedded in paraffin. For immunohistochemistry, 8μm sections were cut from two coronal slices at the level of the optic chiasm and hippocampus from the right hemisphere. Two coronal slices (5mm thick) from the right hemisphere at the level of the optic chiasm and hippocampus were embedded in paraffin and cut into 8μm sections. Prior to immunohistochemistry staining, sections were dehydrated in xylene and rehydrated in graded ethanol solution (100-70%)

Transferase-mediated biotinylated d-UTP nick end-labelled (TUNEL) was used to assess cell death over 8 regions of the brain from both sections. Slices were treated with 3% hydrogen peroxide followed by pre-digestion with protease K (Promega, Southampton, UK) and incubated in TUNEL solution for 2 hours (Roche, Burgess Hill, UK). Slices were then incubated in avidin-biotinylated horseradish peroxidase complex (ABC, Vector Laboratories) followed by diaminobenzidine/H_2O_2 (Sigma) with $CoCl_2$ and $NiCl_2$. A haematoxylin eosin counter stain was applied and slices were mounted on coverslips with DPX. TUNEL-positive cells were counted in 3 fields at X40 magnification for each of the 8 regions of the brain at 2 levels using QuPath (v0.4.4). TUNEL counts were averaged for each subject and for each brain region across all fields and then scaled up to cells per mm^2.

The remaining immunohistochemistry was performed using Roche Ventana Discovery XT system with the following protocol:

	Pretreatment	Primary Antibody	Secondary Antibody	DAB
Olig2	Ventana CC1 (Cell Conditioning Conditioner 1)	Rabbit anti-Olig2, Millipore, AB9610 Conc 1:100 Incubation 4 hours	Goat anti-Rabbit Abcam, ab207995 Conc 1:200 Incubation 32 mins	DAB Map kit (Roche 760-124)
Iba1	Ventana CC1 (Cell Conditioning Conditioner 1)	Rabbit anti-Iba1, Wako, 019-19741 Conc 1:250 Incubation 4 hours	Goat anti-Rabbit Abcam, ab207995 Conc 1:200 Incubation 60 mins	DAB Map kit (Roche 760-124)
NeuN	Ventana CC1 (Cell Conditioning Conditioner 1)	Mouse anti-NeuN, Millipore, MAB377 Conc 1:2000 Incubation 32 mins	Rabbit anti-mouse, Abcam, ab98668 Conc 1:100 Incubation 60 mins	DAB Map kit (Roche 760-124)

A Haematoxylin counterstain (Roche counterstain kit) was applied for 8 mins. Slides were dehydrated in graded alcohol and mounted on DAPI aqueous mounting media (Vector Labs). Slides were scanned using Nanozoomer S60 (Hamamatsu) as brightfield with maximal resolution (40x). Similar to TUNEL, NeuN, Iba1 and Olig2 positive cells were counted in 3 fields at x40 magnification for each region using QuPath, then averaged out per region and scaled up to cells per mm^2. GFAP % area positivity was deduced by comparing the area of positive GFAP staining using the threshold tool on QuPath with the total area assessed.

1.3 Plasma Cytokines

The parameters for the cytokine assays were as follows:

	LLOQ (pg/mL)	ULOQ (pg/mL)
TNF-a	0.3	1160
IL-1ra	12.8	14440
IL-4	62.0	16750
IL-6	7.1	7065
IL-10	7.5	8372

2 Azithromycin Pharmacokinetics (PK) studies

2.1 Dose-finding PK study

The azithromycin (AZI) pilot study aimed to determine the azithromycin dose required in the newborn piglet to achieve the cytoprotective levels observed by Barks et al. [9]. In the neonatal rat study by Barks et al. (2019) [9], azithromycin at 45mg/kg 2h after HI, followed by two doses of 22.5mg/kg at 24 and 48h, was associated with the best cytoprotection. This achieved whole blood C_{max} ~6-10mg/L and brain azithromycin concentrations of ~1.5-2.0

mg/kg at 48h and 2-3mg/kg at 65h. Allometric scaling suggested that 45mg/kg in the rat is equivalent to 12mg/kg in the piglet. The two azithromycin dosing regimens were assessed:

1. Low AZI dose: 12mg/kg loading dose over 1h + 6mg/kg at 24h and 48h (n=3)
2. High AZI dose: 24mg/kg loading dose over 1h + 12mg/kg at 24h and 48h (n=2)

The azithromycin PK profile fits a two-compartment model (See **Figure S1**). The mean Cmax (±SD) in the plasma was 1.49 ±0.42 mg/L with an AUC_{24} of 4.2 (95% CI 2.99 to 5.40) mg/L*h following the 12mg/kg loading dose. The subsequent 6mg/kg maintenance dose led to a Cmax of 0.87 ±0.43 mg/L and AUC_{48} of 8.78 (95% CI 7.08 to 10.48) mg/L*h. Brain tissue AZI concentration at 48h was 0.80 ±0.26 mg/kg from 1 animal. At high dose, AZI 24mg/kg loading was associated with Cmax of 4.31 ±1.31 mg/L and AUC_{24} of 9.27 (95% CI 7.6 to 10.9) mg/L*h. The subsequent dose of 12mg/kg led to a Cmax of 0.98 ±0.25 mg/L and AUC_{48} of 15.12 (95% CI 12.94 to 17.29) mg/L*h.

An important consideration is the difference in the measurement of azithromycin levels between the piglets (plasma) compared to the rat studies (whole blood). Azithromycin accumulates in monocytes, leucocytes and fibroblasts and is stored in lysosomes, contributing to higher levels in whole blood compared to the plasma compartment [11]. Assuming a plasma: whole blood azithromycin concentration ratio of 1:3 as observed in the rat (unpublished, Barks et al.), the whole blood target of 6-10mg/L is equivalent to plasma levels of 2-3 mg/L. The results from the piglet pilot studies suggest that 12mg/kg was insufficient to achieve the extrapolated therapeutic levels in the plasma (~1.5mg/L), which were two-fold lower than target. Furthermore, brain tissue azithromycin concentration (0.80mg/kg) was also two-fold lower (albeit from one animal only). While the brain tissue levels in the high dose azithromycin of 24mg/kg were unavailable, plasma C_{max} levels (~4mg/L) were above the extrapolated target plasma therapeutic levels.

An azithromycin dose of 20mg/kg every 24h was selected for the study based on the following:

- Based on the pilot PK study, 12mg/kg dosing was insufficient to achieve the target therapeutic levels.
- Unpublished data in the sheep model of HI (Maltepe et al., University of California, San Francisco) suggested dosing with 10mg/kg/d was not neuroprotective.
- To ensure clinical translational relevance, intravenous azithromycin 20mg/kg every 24h has been safely administered to preterm infants in several clinical studies [12, 13]. No hypertrophic pyloric stenosis or QT interval prolongation was reported, and no increased risk of gastrointestinal complications.

2.2 Population PK Analysis

Population PK modeling was performed using MonolixSuite 2024R1 (Lixoft SAS, MonolixSuite 2024R1, Antony, France) with the stochastic approximation expectation–maximization (SAEM) algorithm. The dataset contained concentration–time data following administration of azithromycin, analyzed using a two-compartment structural model with first-order elimination. Interindividual variability was modeled exponentially on clearance (Cl), central volume (V1), intercompartmental clearance (Q), and peripheral volume (V2). A combined (proportional + additive) residual error model was applied and modified to identify the optimal error model. Model parameters were estimated using stochastic approximation,

and standard errors were derived from Fisher information matrix-based approximations. Model performance was assessed by examination of diagnostic goodness-of-fit plots (observed vs. predicted concentrations, residual distributions), individual fits, and a visual predictive check (VPC). The final model was selected based on parameter precision, biological plausibility, and fit statistics, including objective function value (OFV) and the Akaike Information Criterion (AIC).

3 Supplementary Tables

Table S1: Physiological parameters between groups

Time Interval	Vehicle	Azithromycin	p value
Rectal Temperature (°C)			
Baseline	37.8 (37.7 to 38)	37.8 (37.6 to 37.9)	0.612
4hr LPS infusion	38.3 (38.1 to 38.4)	38.1 (37.9 to 38.2)	0.047
0-1hr	38.1 (37.9 to 38.2)	38 (37.8 to 38.1)	0.317
1-24hr	38 (37.9 to 38.2)	38 (37.9 to 38.1)	0.699
24-48hr	38 (37.9 to 38.2)	38 (37.9 to 38.1)	0.975
48-65hr	38 (37.9 to 38.2)	38 (37.9 to 38.1)	0.764
Heart Rate (beats per minute)			
Baseline	143 (133 to 154)	147 (137 to 157)	0.608
4hr LPS infusion	165 (155 to 176)	170 (160 to 180)	0.498
0-1hr	175 (165 to 185)	180 (170 to 190)	0.493
1-24hr	188 (178 to 199)	184 (174 to 194)	0.494
24-48hr	159 (149 to 169)	150 (140 to 160)	0.207
48-65hr	139 (129 to 150)	129 (119 to 139)	0.140
Mean Arterial Blood Pressure (mmHg)			
Baseline	50.5 (47.6 to 53.4)	50.4 (47.6 to 53.2)	0.956
4hr LPS infusion	49.3 (46.3 to 52.2)	48.8 (45.9 to 51.6)	0.808
0-1hr	38.8 (35.9 to 41.8)	42 (39.2 to 44.8)	0.129
1-24hr	46 (43 to 48.9)	47.1 (44.3 to 49.9)	0.578
24-48hr	49.8 (46.8 to 52.7)	51.1(48.3 to 53.9)	0.512
48-65hr	50.5 (47.6 to 53.4)	50.8 (48 to 53.6)	0.885
Vaso-inotropic Score			
0-1h	0 (0 to 0.87)	1.40 (0.56 to 2.23)	
1-24hr	5.8 (0.33 to 11.19)	6.5 (1.28 to 11.72)	
24-48hr	2.58 (0.14 to 5.02)	0.47 (0 to 2.82)	
48-65hr	0.31 (0 to 0.91)	0.35 (0 to 0.93)	
pH			
Baseline	7.45 (7.41 to 7.5)	7.44 (7.39 to 7.49)	0.658
4hr LPS infusion	7.41 (7.36 to 7.46)	7.42 (7.37 to 7.47)	0.795
0-1hr	7.3 (7.25 to 7.35)	7.28 (7.23 to 7.33)	0.664
1-24hr	7.48 (7.43 to 7.52)	7.43 (7.39 to 7.48)	0.212
24-48hr	7.46 (7.42 to 7.51)	7.46 (7.41 to 7.5)	0.795
48-65hr	7.46 (7.41 to 7.51)	7.45 (7.4 to 7.5)	0.784
pCO_2 (kPa)			
Baseline	6.4 (5.8 to 7.1)	6.3 (5.6 to 6.9)	0.686
4hr LPS infusion	6.2 (5.6 to 6.8)	5.7 (5.1 to 6.4)	0.339
0-1hr	6 (5.3 to 6.6)	5.6 (4.9 to 6.2)	0.431

1-24hr	5.3 (4.7 to 5.9)	5.2 (4.6 to 5.8)	0.898
24-48hr	5.4 (4.7 to 6)	5.2 (4.6 to 5.8)	0.699
48-65hr	5.2 (4.6 to 5.8)	5.4 (4.8 to 6)	0.711
Base Excess (mEq/L)			
Baseline	7.6 (5.4 to 9.8)	5.7 (3.6 to 7.8)	0.212
4hr LPS infusion	4.8 (2.6 to 7)	3.3 (1.2 to 5.3)	0.297
0-1hr	-4.6 (-6.8 to -2.4)	-6.9 (-9.1 to -4.7)	0.14
1-24hr	5.6 (3.5 to 7.8)	3.7 (1.6 to 5.7)	0.181
24-48hr	5 (2.9 to 7.2)	3.5 (1.4 to 5.6)	0.296
48-65hr	3.9 (1.8 to 6.1)	3.9 (1.8 to 5.9)	0.950
Lactate (mmol/L)			
Baseline	2.8 (1.2 to 4.3)	3.3 (1.7 to 4.8)	0.676
4hr LPS infusion	4.5 (3 to 6.1)	5.5 (4 to 7)	0.436
0-1hr	10.5 (8.9 to 12.1)	11.4 (9.8 to 13.1)	0.430
1-24hr	3.5 (1.9 to 5.1)	4.2 (2.7 to 5.7)	0.566
24-48hr	2 (0.4 to 3.6)	2 (0.5 to 3.5)	0.999
48-65hr	1.3 (-0.2 to 2.9)	1.6 (0.1 to 3.1)	0.805
Glucose (mmol/L)			
Baseline	5.9 (4.6 to 7.2)	6.2 (5 to 7.5)	0.531
4hr LPS infusion	5.8 (4.5 to 7.1)	5.9 (4.6 to 7.2)	0.852
1-24hr	5 (3.7 to 6.3)	5.3 (4 to 6.6)	0.570
24-48hr	5.9 (4.5 to 7.2)	4.8(3.6 to 6.1)	0.062
48-65hr	5.5 (4.1 to 6.8)	5 (3.8 to 6.3)	0.434
Potassium (mmol/L)			
Baseline	4.3 (3.7 to 4.9)	4.1 (3.5 to 4.7)	0.627
4hr LPS infusion	4.3 (3.7 to 5)	4.4 (3.8 to 4.9)	0.990
1-24hr	5.9 (5.3 to 6.5)	5.8 (5.2 to 6.4)	0.777
24-48hr	5.7 (5.1 to 6.3)	5.1 (4.5 to 5.7)	0.113
48-65hr	5.5 (4.9 to 6.1)	4.4 (3.8 to 5)	**0.003**
Creatinine			
Baseline	66 (29.6 to 102.4)	64.5 (30.6 to 98.3)	0.939
24-48hr	193.2 (158 to 228.4)	190.5 (153 to 228)	0.916
48-65hr	240.2 (203.8 to 276.6)	172.5 (138.7 to 206.4)	**0.012**

Table S2: Exploratory sex-stratified MRS and aEEG/EEG data. The study was not powered for sex-specific effects; descriptive estimates are shown only.

Outcome	Treatment Difference IA-HI-AZI vs IA-HI Veh Mean (95% CrI)		
	Combined	Male	Female
**BGT Lac/NAA**	-0.143 (-0.456, 0.170)	-0.329 (-0.974, 0.316)	0.054 (-0.221, 0.328)
**WM Lac/NAA**	-0.109 (-0.576, 0.356)	-0.215 (-0.937, 0.505)	-0.035 (-0.736, 0.661)
**aEEG/EEG**	0.053 (0.006; 0.099)	0.038 (-0.050; 0.123)	0.062 (-0.008; 0.131)

Table S3: Iba1 high-magnification sensitivity analysis. Data shown include regional and overall Iba1 ramification index outcomes quantified at 40× (primary analysis) and 60× (sensitivity analysis) across the same two coronal slices. Bayesian mean treatment differences, 95% credible intervals (CrI) and probabilities of superiority [Pr(sup)] are presented to assess whether restricting quantification to clearly viable microglia at higher magnification yields conclusions consistent with the primary analysis.

Region	x40 Magnification (Primary Analysis)		x60 Magnification (Sensitivity Analysis)	
	**Mean Difference [95% CrI]**	**$Pr_{(sup)}$**	**Mean Difference [95% CrI]**	**$Pr_{(sup)}$**
cCTx	-0.14 [-0.39; 0.11]	13.60%	0 [-0.5; 0.5]	50.70%
sCTx	0.10 [-0.15; 0.36]	78.30%	0.5 [0; 1.1]	**97.50%**
Hip	0.00 [-0.33; 0.34]	51.10%	0.1 [-0.4; 0.6]	66.00%
pvWM	0.20 [-0.04; 0.45]	**94.70%**	0.1 [-0.4; 0.6]	64.40%
IC	0.24 [-0.02; 0.49]	**96.70%**	0.2 [-0.4; 0.7]	72.40%
Caud	0.35 [0.09; 0.61]	**99.50%**	0.7 [0.2; 1.2]	**99.70%**
PTMN	0.19 [-0.07; 0.45]	92.20%	0.6 [0.1; 1.1]	**98.70%**
Thalamus	0.33 [0.08; 0.58]	**99.50%**	0.5 [0; 1]	**96.70%**
**Overall**	**0.17 [0.05; 0.30]**	**99.60%**	**0.41 [0.05; 0.71]**	**98.80%**

Table S4: Comparison between frequentist and Bayesian analysis. Data shown include a sample of outcome measures from the main analysis, with Bayesian mean treatment differences, 95% credible intervals (CrI) and probabilities of superiority [$Pr_{(sup)}$] presented alongside the corresponding frequentist effect estimates, 95% confidence intervals (CI) and p values. Statistical significance is indicated by * where $p < 0.05$ or $Pr_{(sup)} \geq 94.8\%$, showing close agreement between the two approaches. Data marked ^ indicate outcomes where $p > 0.05$, but the Bayesian analysis suggests a potential biological signal.

Outcomes	Bayesian Analysis		Frequentist Analysis	
	Mean Difference [95% CrI]	$Pr_{(sup)}$	Mean Difference (95% CI)	P value
**BGT Lac/NAA**	-0.143 [-0.456; 0.170]	**82.7%^**	-0.141 (-0.435, 0.153)	0.329
**WM Lac/NAA**	-0.109 [-0.576; 0.356]	68.5%	-0.208 (-0.542, 0.327)	0.611
**aEEG/EEG** Overall	0.053 [0.006; 0.099]	**98.6%***	0.322 (0.003, 0.061)	**0.040***

**aEEG/EEG**<br>7-12h	-0.362<br>[-0.716; -0.009]	2.30%	-0.306<br>(-0.082, 0.694)	0.115
**aEEG/EEG**<br>13-18h	-0.145<br>[-0.695; 0.404]	29.40%	-0.047<br>(-0.446, 0.540)	0.845
**aEEG/EEG**<br>19-24h	-0.071<br>[-0.720; 0.579]	41.20%	-0.095<br>(-0.432, 0.622)	0.713
**aEEG/EEG**<br>25-30h	0.166<br>[-0.396; 0.728]	72.90%	0.211<br>(-0.625, 0.203)	0.294
**aEEG/EEG**<br>31-36h	0.182<br>[-0.234; 0.596]	**81.50%^**	0.138<br>(-0.427, 0.151)	0.328
**aEEG/EEG**<br>37-42h	0.230<br>[-0.172; 0.630]	**87.90%^**	0.179<br>(-0.470, 0.111)	0.207
**aEEG/EEG**<br>43-48h	0.225<br>[-0.291; 0.742]	**81.50%^**	0.149<br>(-0.571, 0.272)	0.469
**aEEG/EEG**<br>49-54h	0.228<br>[-0.321; 0.780]	**80.20%^**	0.226<br>(-0.609, 0.156)	0.228
**aEEG/EEG**<br>55-60h	0.461<br>[-0.040; 0.961]	**96.60%***	0.380<br>(-0.778, 0.017)	***0.059***
**NeuN**<br>Overall	0.36<br>[0.01; 0.72]	**97.8%***	0.359<br>(0.051, 0.668)	**0.032***
**NeuN**<br>cCTx	0.6<br>[0; 1.2]	**97.7%***	0.629<br>(0.024, 1.234)	**0.042***
**NeuN**<br>sCTx	0.1<br>[-0.5; 0.8]	68.4%	0.153<br>(-0.455, 0.761)	0.619
**NeuN**<br>Hip	0.2<br>[-0.6; 1]	69.2%	0.203<br>(-0.575, 0.980)	0.608
**NeuN**<br>pvWM	0.6<br>[0; 1.3]	**98.0%***	0.645<br>(0.040, 1.250)	**0.037***
**NeuN**<br>IC	0.3<br>[-0.3; 1]	**86.7%^**	0.353<br>(-0.249, 0.955)	0.249
**NeuN**<br>Caud	0.7<br>[0.1; 1.3]	**98.9%***	0.723<br>(0.121, 1.324)	**0.019***
**NeuN**<br>PTMN	0.4<br>[-0.2; 1]	**90.8%^**	0.420<br>(-0.182, 1.022)	0.170
**NeuN**<br>Thal	0.6<br>[0; 1.2]	**97.0%***	0.582<br>(-0.020, 1.184)	***0.058***

4 Supplementary Figures

Figure S1 PK profile in pilot study to assess two doses: low dose (12mg/kg loading dose over 1h + 6mg/kg at 24h and 48h) and high dose (24mg/kg loading dose over 1h + 12mg/kg at 24h and 48h)

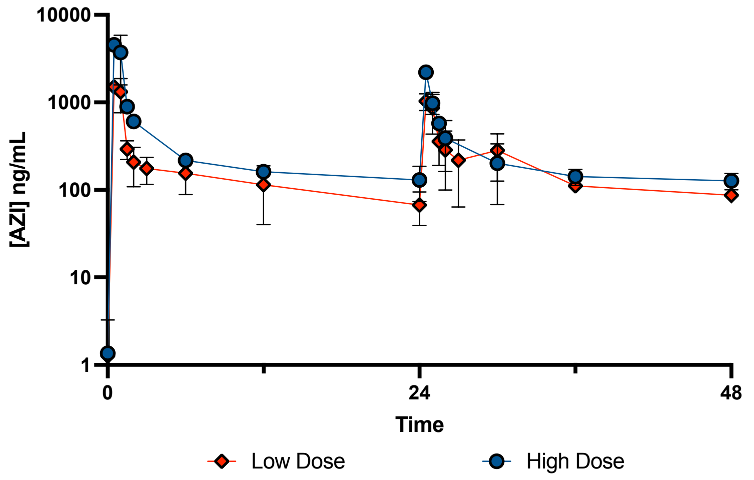

Figure S2: Pharmacokinetics of Azithromycin. Data shown include the mean plasma (±SD) azithromycin levels (mg/L) (A) and goodness of fit plots, observed against the individual predicted azithromycin plasma concentration (B), weighted residuals against time and azithromycin prediction (C), and the visual predictive check (D).

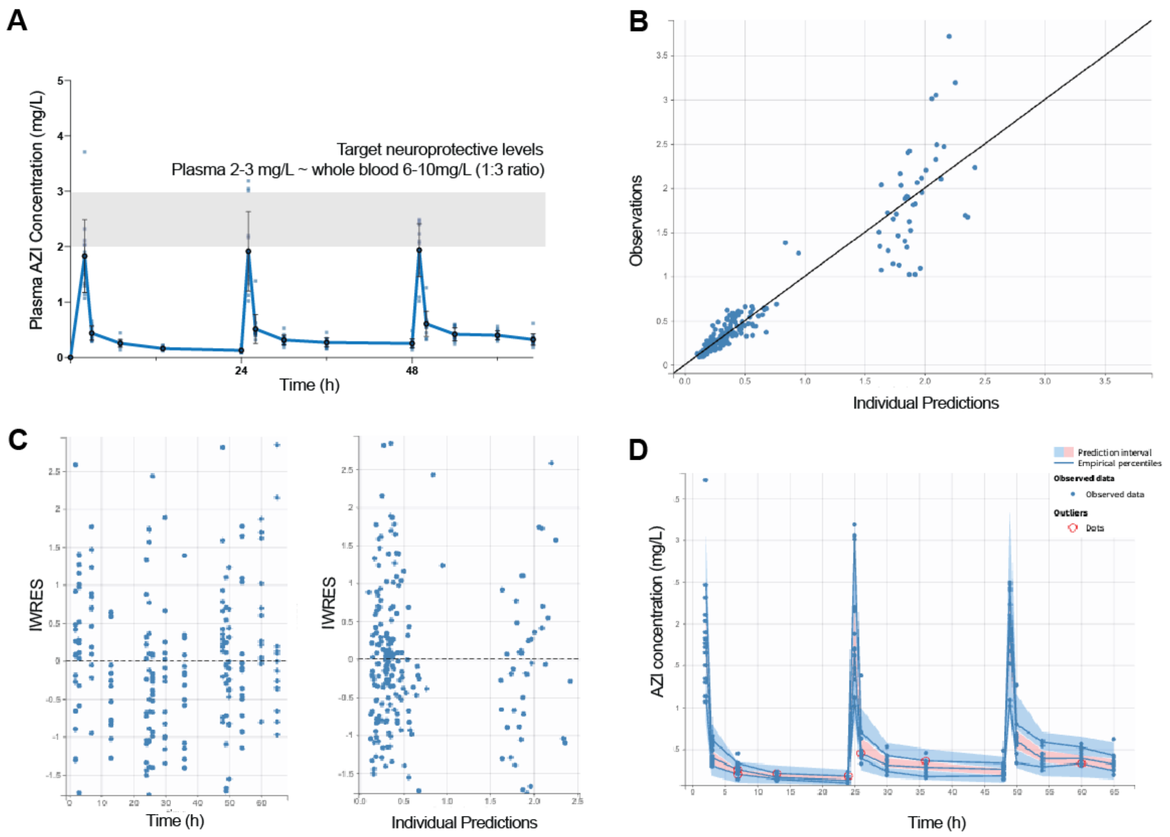

Figure S3: Haematology Markers Comparison of (mean ±SEM) white cell count (A), neutrophils (B), eosinophils (C), lymphocytes (D), platelet (E) and systemic inflammatory response index (F) between groups. A two-way ANOVA model by time, group and time*group with a random individual for repeated measures was applied. Statistical significance comparing HI-AZI vs HI-vehicle was assessed using an ANOVA model where * indicates p<0.05 between groups.

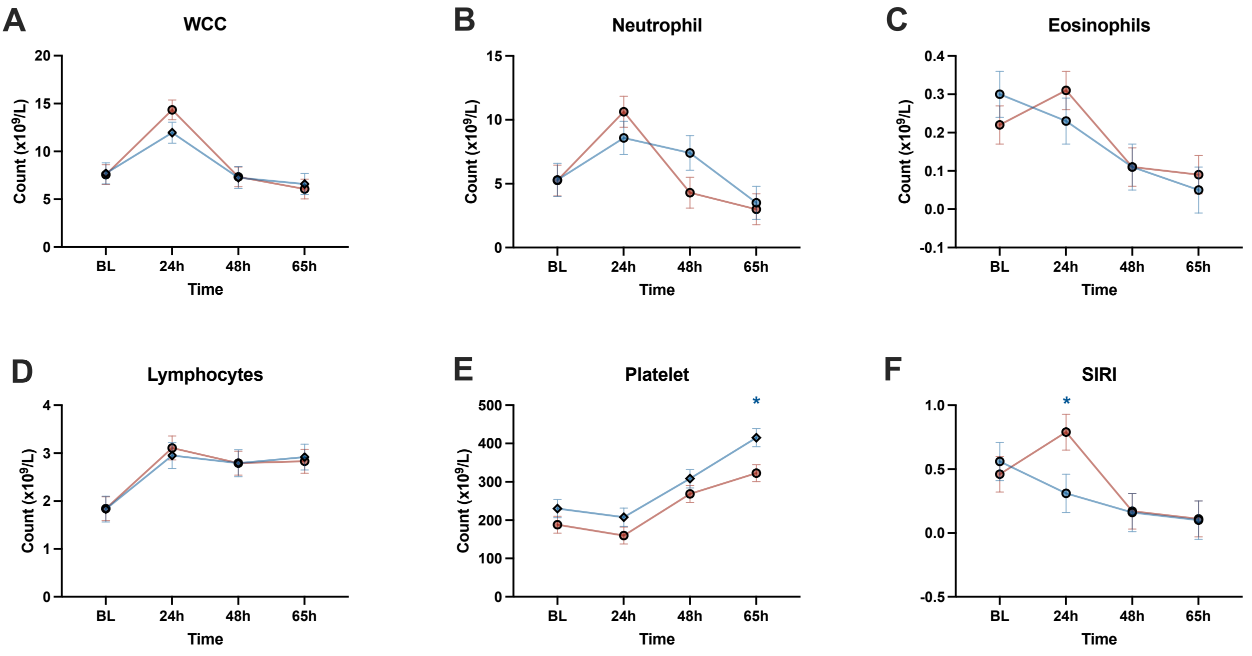

Figure S4: Plasma Cytokine Response. Plasma IL-4, IL-6, IL-10 and IL-12 were measured and compared between treatment group at baseline, end of HI, 24, 48 and 60h. The data was analysis using a two-way ANOVA model following log_{10} transformation by time, group and group*time with a random individual for repeated measures. No significant differences were observed between groups.

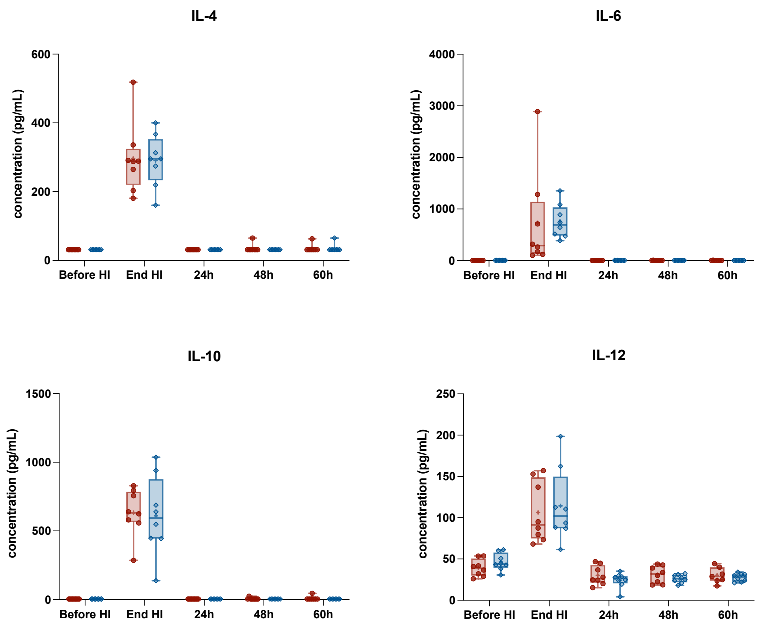

Figure S5: Pilot high-dose 60mg/kg study. Two piglets received 3 doses of 60mg/kg over 1h at 1h, 24h and 48h after IA-HI. The PK profile is shown in A with peak azithromycin levels of 7-10mg/L, achieving the target range. However, in both animals, high-dose infusion was associated significant fall in MABP within 30 mins of infusion (B) and increased inotropic support (C).

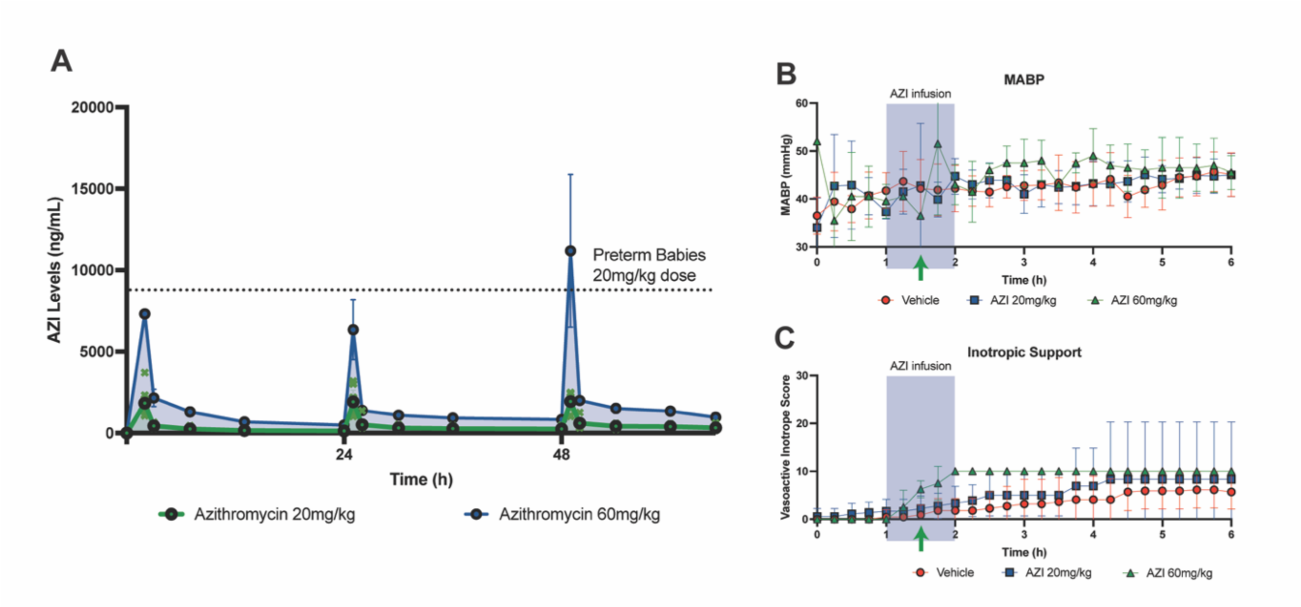

Supplement: Supplementary file 1 [file str-57-1362-s001.pdf]
