# Supplementary material for: Postnatal Azithromycin Is Neuroprotective and Anti-Inflammatory in a Piglet Model of Hypoxic-Ischemic Encephalopathy
Source: Stroke. 2026 Feb 13;57(5):1362–75. doi: 10.1161/STROKEAHA.125.054318 (PMC13117526; doi:10.1161/STROKEAHA.125.054318)
Supplement: Supplementary file 3 [file str-57-1362-s003.pdf]

Major Resources Table

In order to allow validation and replication of experiments, all essential research materials listed in the Methods should be included in the Major Resources Table below. Authors are encouraged to use public repositories for protocols, data, code, and other materials and provide persistent identifiers and/or links to repositories when available. Authors may add or delete rows as needed.

Additional Species Used in This Study (if applicable)

Strain	Vendor or Source	Background Strain	Sex	Persistent ID / URL
Large White Piglet crossed with Landrace (Sus scrofa domesticus)	Commercial Farm, Rollins Farm Ltd - Cambridgeshire	N/A	Male and Female	N/A

ARRIVE GUIDELINES

The ARRIVE guidelines (https://arriveguidelines.org/) are a checklist of recommendations to improve the reporting of research involving animals. Key elements of the study design should be included below to better enable readers to scrutinize the research adequately, evaluate its methodological rigor, and reproduce the methods or findings.

Study Design

Groups	Sex	Age	Number (prior to experiment)	Number (after termination)	Littermates (Yes/No)	Other description
Group 1 (Control)	Male and Female	<48h old	13	12	No	This group received a vehicle control after IA-HI<br><br>1 animal died at 50h due to refractory hyperkalaemia secondary to severe renal failure, prior to MRS acquisition
Group 2 (Azithromycin)	Male and Female	<48h old	14	13	No	This group received IV AZI 20mg/kg at 1h, 24h and 48h after IA-HI<br><br>1 animal was excluded after HI as the injury was too severe, falling outside the standard insult parameter range.

Sample Size

Lac/NAA was used to power the sample size calculations given its strong translational relavence as a biomarker to predict 2 year neurodevelopmental outcomes in babies with moderate-severe HIE in the clinical setting, correlating with immunohistochemistry outcomes in our piglet model.

DOI [to be added]

In previous piglet studies, we observed a treatment difference of 0.5–0.77 $\log_{10}$ reduction in ^{1}H MRS lactate/N-acetylaspartate ratio (Lac/NAA) with a standard deviation of 0.34-0.42. Using a pessimistic prediction of 0.5 Log_{10} unit reduction in basal ganglia and thalamus (BGT) Lac/NAA and a standard deviation of 0.4, 12 piglets per group were required to achieve 80% power at 5% type-I error rate.

Inclusion Criteria

Piglets were entered into the preclinical study if the following criteria were met:

1. Piglets were healthy on visual inspection on arrival with no evidence of pyrexia (temperature >39.5°C, diarrhoea, or significant external injury)
2. No complications (namely hypoxia or severe metabolic acidosis) during and following surgical preparation
3. Normal (continuous normal voltage – see aEEG section) cerebral background activity on aEEG after surgery
4. Survival after HI insult
5. No recovery of aEEG (aEEG remains isoelectric) within 1h after HI

Exclusion Criteria

Piglets were excluded from the study if any of the following criteria were met:

1. Evidence of aEEG/EEG recovery within 1h of insult indicating mild HI injury
2. Cardiogenic shock not responsive to neonatal intensive care management leading to secondary brain injury
3. Evidence of complete lack of perfusion on cerebral NIRS (extreme outliers of $rSaO_2$ values) during the experiment. This indicates evolving severe cerebral oedema ± coning; piglets do not have open fontanelles unlike the newborn baby therefore this condition would not be translationally relavent

Randomization

At the conclusion of of the acute IA-HI insult and piglets were resuscitated, piglets were randomised using a blinded computer-generated random allocation (Microsoft Excel) process at 1h after insult to receive either intravenous (i) vehicle (IA-HI-vehicle) or (ii) azithromycin (IA-HI-AZI). Block randomization was performed stratified by sex to ensure equal sex distribution at interim analysis (minimum of 6 animals per sex)

Blinding

Treatment allocation did not occur prior to HI insult. While it was not feasible to blind investigators to the treatment arms during the treatment period and experimentation, the analysis of neurological outcomes (aEEG/EEG, MRS, Immunohistochemistry) were performed with assessors blinded to the treatment group. Individual piglets were identified by a unique participant ID number (LWP XXX) for the purpose of outcome assessment, and unblinding occurred following statistical analysis.

DOI [to be added]
